# Bayesian network analysis of factors influencing type 2 diabetes, coronary heart disease, and their comorbidities

**DOI:** 10.1186/s12889-024-18737-x

**Published:** 2024-05-08

**Authors:** Danli Kong, Rong Chen, Yongze Chen, Le Zhao, Ruixian Huang, Ling Luo, Fengxia Lai, Zihua Yang, Shuang Wang, Jingjing Zhang, Hao Chen, Zhenhua Mai, Haibing Yu, Keng Wu, Yuanlin Ding

**Affiliations:** 1 Department of Epidemiology and Medical Statistics, School of Public Health, Guangdong Medical University, Dongguan, 523808 Guangdong China; 2 Department of Infection Control, Ankang Hospital of Traditional Chinese Medicine, Ankang, 725000 Shaanxi China; 3 Department of Gastroenterology, Affiliated Hospital of Guangdong Medical University, Zhanjiang, 524002 Guangdong China; 4 School of Public Health and Emergency Management, South University of Science and Technology of China, Shenzhen, 518055 Guangdong China; 5 Department of Critical Care Medicine, Affiliated Hospital of Guangdong Medical University Zhanjiang, Zhanjiang, 524001 China; 6 Department of Cardiology, Affiliated Hospital of Guangdong Medical University, Zhanjiang, 524002 Guangdong China

**Keywords:** Type 2 diabetes, Coronary heart disease, Comorbidity, Influencing factors, BN

## Abstract

**Objective:**

Bayesian network (BN) models were developed to explore the specific relationships between influencing factors and type 2 diabetes mellitus (T2DM), coronary heart disease (CAD), and their comorbidities. The aim was to predict disease occurrence and diagnose etiology using these models, thereby informing the development of effective prevention and control strategies for T2DM, CAD, and their comorbidities.

**Method:**

Employing a case-control design, the study compared individuals with T2DM, CAD, and their comorbidities (case group) with healthy counterparts (control group). Univariate and multivariate Logistic regression analyses were conducted to identify disease-influencing factors. The BN structure was learned using the Tabu search algorithm, with parameter estimation achieved through maximum likelihood estimation. The predictive performance of the BN model was assessed using the confusion matrix, and Netica software was utilized for visual prediction and diagnosis.

**Result:**

The study involved 3,824 participants, including 1,175 controls, 1,163 T2DM cases, 982 CAD cases, and 504 comorbidity cases. The BN model unveiled factors directly and indirectly impacting T2DM, such as age, region, education level, and family history (FH). Variables like exercise, LDL-C, TC, fruit, and sweet food intake exhibited direct effects, while smoking, alcohol consumption, occupation, heart rate, HDL-C, meat, and staple food intake had indirect effects. Similarly, for CAD, factors with direct and indirect effects included age, smoking, SBP, exercise, meat, and fruit intake, while sleeping time and heart rate showed direct effects. Regarding T2DM and CAD comorbidities, age, FBG, SBP, fruit, and sweet intake demonstrated both direct and indirect effects, whereas exercise and HDL-C exhibited direct effects, and region, education level, DBP, and TC showed indirect effects.

**Conclusion:**

The BN model constructed using the Tabu search algorithm showcased robust predictive performance, reliability, and applicability in forecasting disease probabilities for T2DM, CAD, and their comorbidities. These findings offer valuable insights for enhancing prevention and control strategies and exploring the application of BN in predicting and diagnosing chronic diseases.

## Introduction

With the continuous improvement of economic level, the acceleration of population aging and the change of people's lifestyle, chronic diseases have become a major public health problem threatening human health. Among them, Diabetes Mellitus (DM) and CAD are two common chronic diseases that seriously endanger human health. Diabetes is a serious chronic metabolic disease, which is the pathological basis of a variety of chronic complications, especially vascular complications. T2DM is the most common, accounting for more than 90% of all diabetic patients [1]. According to the "China Type II Diabetes Prevention and Control Guidelines (2020 edition)" data show that the prevalence of diabetes in people over 18 years old in China was 10.4% in 2013, and has risen to 11.2% in 2017, with a total population of more than 110 million, and research estimates that the number of people with diabetes will reach 130 million by 2030, and its prevention and control situation is becoming increasingly severe [2, 3]. Coronary heart disease, short for coronary heart disease, is the most common type of organ damage caused by atherosclerosis. Data from the China Health Statistical Yearbook 2020 show that the mortality rate of coronary heart disease continues to rise since 2012, and the death caused by cardiovascular disease ranks first in the total cause of death of urban and rural residents in China, higher than tumor and other diseases, and its harm should not be underestimated [4, 5].

T2DM and CAD are closely related. T2DM and CAD are closely related, with the coexistence of T2DM and CAD representing a prevalent comorbidity. These condi8tions share a common etiological chain, grounded in both genetic and metabolic factors [6–8]. People with diabetes have two to four times the risk of developing cardiovascular disease than people without diabetes, and the American Heart Association states that "diabetes is a cardiovascular disease" [9, 10]. The Chinese Heart Survey also found that 53% of patients with cardiovascular disease had diabetes. As the main complication of diabetic patients, cardiovascular disease is the leading cause of death in diabetic patients, accounting for more than 70% of the total number of diabetic patients [11]. Moverover, chronic diseases such as T2DM and CAD share a common chain of etiology. In addition to unchangeable influencing factors such as age, gender and heredity, risk factors such as bad lifestyle and unhealthy eating habits can be interfered with, which is also the main entry point for the prevention and treatment of chronic diseases. Therefore, to explore the related influencing factors of T2DM, CAD and their comorbidities, it is of great significance for its early prevention, timely diagnosis and treatment.

For the high incidence and poor prognosis of T2DM and CAD, correct prediction and timely diagnosis are of great significance in clinical medicine. Most of the previous researches on the influencing factors of such diseases used traditional logistic regression, or machine learning methods such as cluster analysis, decision trees and artificial neural networks. Due to the limitation of its principle, logistic regression requires the independence of variables. At the same time, the logistic regression model must know the state of each variable in order to predict the outcome probability. However, most chronic non-communicable diseases, especially those represented by T2DM and CD, are the result of complex multi-factor interaction, and each factor is not independent of the other. In addition, in most cases, patients' clinical information is incomplete. For example, patients may deliberately conceal some information, resulting in incomplete data. For incomplete data, logistic regression cannot predict the corresponding probability, which exposes certain limitations of traditional statistical methods. However, machine learning methods such as decision trees and artificial neural networks will inevitably have problems such as complicated results, difficult to quantify and inflexible when dealing with complex relationships of diseases.

BN is a directed acyclic graph representing probabilistic dependencies between variables, consisting of nodes and directed edges, where nodes represent random variables and directed edges represent probabilistic dependencies between variables. Each node has a corresponding Conditional probability distribution table (CPT), which is used to represent the probabilistic dependency between the node variable and the parent node variable [12]. BN consists of two parts: structure learning and parameter learning. Structure learning refers to the problem of how to determine the network structure when the network structure is unknown [13]. In recent years, with the continuous in-depth study of Bayesian networks by scholars at home and abroad, many kinds of Bayesian network structure learning algorithms have been proposed. Tabu Search (TS) algorithm [14] is a sub-heuristic random search algorithm. It is a global intelligent optimization algorithm, which mainly simulates human memory function, and obtains the global optimal solution by gradually searching the local neighborhood. In order to avoid falling into local optimization and repeated iteration, TS algorithm adopts a tabu table to record and select the mobile search process of solutions. When the optimal solution is already in the tabu table, the solution will be automatically abandoned [15] to avoid repeated search. It can not only find the local optimal solution in the search space, but also jump out of the local search to get the global optimal solution. Previous studies have verified that the Bayesian network model constructed by TS algorithm has better fitting results than other algorithms [16, 17]. The main purpose of BN parameter learning is to determine the conditional probability distribution among nodes in the network by using prior knowledge based on the existing network structure [18]. For the learning of complete data sets, commonly used parameter learning methods mainly include maximum likelihood estimation(MLE) [19], Bayesian estimation(BE) [20], etc., while for missing data sets, approximate algorithms are generally adopted, such as expectation maximization algorithm(EM) [21]. BN combines the advantages of probability theory and graph theory well. It not only utilizes the intuitionism of graph theory to describe the direct and indirect relationship between variables, but also makes use of the structure of the network to calculate probabilities, thus reducing the difficulty of reasoning. Compared with other algorithm models, The advantage of BN lies in their ability to reveal more information in data. In recent years, numerous scholars have applied Bayesian networks to investigate factors influencing diseases. For instance, Lou Shuping et al. [22] utilized Bayesian network analysis to construct a model with obesity influencing factors as nodes, depicting influencing relationships as paths, and assigning influencing probabilities as path parameters. Their aim was to qualitatively and quantitatively analyze the correlation and dependence of sleep time and related lifestyle on diseases. Similarly, Wang Bin et al. [23] established a Bayesian network model to identify predictors of severe hand, foot, and mouth disease, and to explore the dependence between corresponding variables. Their research demonstrates that Bayesian networks exhibit promising predictive performance and application potential. Ma Yang et al. [24] also employed the Bayesian network model to visualize the multi-level relationship among various factors affecting the length of hospital stay in trauma patients. They utilized conditional probabilistic reasoning to predict the likelihood of long hospital stays in patients under different circumstances, aiming to enhance the prognosis of trauma patients and reduce medical costs. Additionally, Moradi et al. [25] explored potential directional relationships between symptoms of major depression using directed acyclic graphs and Bayesian networks. They revealed the highest centrality of depressive mood symptoms and suggested that body weight and appetite symptoms exhibited the strongest connection in the network. Their findings will facilitate tailored interventions for patients with major depression. Furthermore, Badawi et al. [26] utilized a Bayesian network mo del to investigate the degree of association between hepatitis C virus infection and cardiovascular disease risk. They discovered that infection was linked to an increased risk of cardiovascular disease and identified variables such as T2DM, hypertension, and age as mediators in this association. The aforementioned studies provide a solid theoretical basis and insights for constructing a Bayesian network model of T2DM, CAD, and their comorbidities."

Therefore, in order to make a more intuitive and comprehensive analysis of the influencing factors of T2DM, CAD and their comorbidities as well as the specific relationships among these factors, this study used BNs to build the disease models of T2DM, CAD and their comorbidities, and carried out disease prediction reasoning and etiology diagnosis. It provides reference for further improving the prevention and control strategies of T2DM, CAD and their comorbidities, and exploring the application of BN in the prediction and diagnosis of chronic diseases.

## Data and methods

### Research object

In this study, data of T2DM, CAD and their comorbidities were collected in the Department of Endocrinology and Cardiology of the Affiliated Hospital and the Second Affiliated Hospital of Guangdong Medical University from July 2021 to December 2022 as the case group, and data of healthy people were collected in the physical examination center during the same period as the control group. This study was approved by the Medical Ethics Committee of the Affiliated Hospital of Guangdong Medical University and the Second Affiliated Hospital, and informed consent was obtained from all subjects and/or their legal guardian(s).

Diagnostic criteria for T2DM: According to the World Health Organization's diagnostic criteria for diabetes in 1999 [27], people with typical diabetes symptoms (polydipsia, polyuria, polydipsia, unexplained weight loss, retinopathy, lower extremity ulcer and other metabolic disorders) should have random blood glucose ≥ 11.1 mmol/L or fasting blood glucose ≥ 7.0 mmol/L. Or glucose tolerance test 2 h blood sugar ≥ 11.1 mmol/L, or a history of diabetes, currently taking hypoglycemic drugs and normal blood sugar, diagnosed as diabetes.

CAD diagnostic criteria [28]: Confirmed by coronary angiography, patients with a history of at least one coronary artery stenosis ≥ 50% or complicated with old myocardial infarction, after percutaneous coronary intervention, or after coronary artery bypass transplantation.

Diagnostic criteria for comorbidities: Simultaneously meeting the T2DM and CAD diagnostic criteria mentioned above.

### Data collection

Data collection mainly includes three parts: questionnaire survey, physical examination and laboratory test.

The questionnaire includes: (1) basic demographic characteristics: age, sex, region, education level, marital status, occupation;(2) family history (FHx) of disease: FHx of DM, FHx of CAD;(3) Living habits and self-care: smoking, drinking, staple food, vegetables, meat, fruits, sweets, exercise, sleep time.

The medical examination includes: Height, weight, waist circumference, hip circumference, Systolic blood pressure (SBP), Diastolic blood pressure (DBP) and heart rate are measured in accordance with a unified method. All surveyors participate in unified training and examination. Only qualified persons may participate in the measurement work.

Laboratory tests include: Fasting blood glucose (FBG), Total cholesterol (TC), Triglyceride (Triglyceride)Determination of TG, High density lipoprotein cholesterol (HDL-C), Low density lipoprotein cholesterol (LDL-C), The biochemical indexes were determined for the first time within 24 h after admission.

### Statistical methods

SPSS 25.0 was used for statistical analysis. The counting data were expressed as frequency and percentage, and the comparison of rates was performed using χ2 test. Measurement data were represented by or M(P25,P75), and comparison between groups was performed by two-independent sample t test or non-parametric test.$\overline{x }\pm s$ Multivariate Logistic regression analysis was carried out using the stepwise method of conditional likelihood ratio.R4.2.0 is used to learn the structure of the BN, maximum likelihood estimation is used to learn the parameters of the BN, and Netica software is used to visualize the predictive reasoning, diagnostic reasoning and sensitivity analysis of Bayesian networks.

## Results

### General description of the research object

A total of 3824 complete data were collected in this study, including 1175 control cases, 726 males (61.79%) and 449 females (38.21%), with an average age of 40.79 ± 14.02 years. There were 1163 T2DM patients, including 635 males (54.60%) and 528 females (45.40%), with an average age of 59.98 ± 12.06 years. There were 982 cases in the CAD group, including 641 males (65.27%) and 341 females (34.73%), with an average age of 67.16 ± 11.75 years. There were 504 patients in the comorbidities group, including 312 males (61.90%) and 192 females (38.10%), with an average age of 67.65 ± 10.38 years. The general description of the research objects is shown in Table 1.

**Table 1:** General description of research objects

Variable	Control (*n* = 1175)	T2DM (*n* = 1163)	CAD (*n* = 982)	Comorbidity (*n* = 504)
**Age**	40.79 ± 14.02	59.98 ± 12.06	67.16 ± 11.75	67.65 ± 10.38
**Sex**				
Male	726 (61.79)	635 (54.60)	641 (65.27)	312 (61.90)
Female	449 (38.21)	528 (45.40)	341 (34.73)	192 (38.10)
**Area**				
Rural	29 (2.47)	447 (38.44)	378 (38.49)	142 (28.17)
Suburban	48 (4.09)	187 (16.08)	114 (11.61)	90 (17.86)
Urban	1098 (93.45)	529 (45.59)	490 (49.90)	272 (53.97)
**Education**				
illiteracy	8 (0.68)	92 (7.91)	95 (9.67)	44 (8.73)
Primary, middle, high, technical secondary school	259 (22.04)	936 (80.48)	812 (82.69)	418 (82.94)
College, bachelor, master degree or above	908 (77.28)	135 (11.61)	75 (7.64)	42 (8.33)
**Marriage**				
Married	853 (72.60)	1099 (94.50)	949 (96.64)	482 (95.63)
Unmarried, divorced, widowed	322 (27.40)	64 (5.50)	33 (3.36)	22 (4.37)
**Occupation**				
Agriculture and forestry	62 (5.28)	368 (31.64)	270 (27.49)	120 (23.81)
Business services	56 (4.77)	166 (14.27)	62 (6.31)	30 (5.95)
Military, national party	785 (66.81)	137 (11.78)	123 (12.53)	61 (12.10)
Students	68 (5.79)	4 (0.34)	1 (0.10)	1 (0.20)
Others	204 (17.36)	488 (41.96)	526 (53.56)	292 (57.94)
**FHx of DM**				
No	1022 (86.98)	938 (80.65)	943 (96.03)	440 (87.30)
Yes	153 (13.02)	225 (19.35)	39 (3.97)	64 (12.70)
**FHx of CAD**				
No	1090 (92.77)	1122 (96.47)	921 (93.79)	466 (92.46)
Yes	85 (7.23)	41 (3.53)	61 (6.21)	38 (7.54)
**Smoke**				
Never	933 (79.40)	866 (74.46)	654 (66.60)	357 (70.83)
Occasion	99 (8.43)	63 (5.42)	71 (7.23)	37 (7.34)
Often	114 (9.70)	196 (16.85)	183 (18.64)	78 (15.48)
Quitting	29 (2.47)	38 (3.27)	74 (7.54)	32 (6.35)
**Drink**				
Never	771 (65.62)	944 (81.17)	781 (79.53)	412 (81.75)
Occasionally	354 (30.13)	133 (11.44)	108 (11.00)	50 (9.92)
Often	36 (3.06)	52 (4.47)	63 (6.42)	22 (4.37)
Quitting	14 (1.19)	34 (2.92)	30 (3.05)	20 (3.97)
**Staple food (liang/day)**				
Never or < 3	626 (52.94)	708 (60.88)	291 (29.63)	193 (38.29)
3–6	489 (41.62)	379 (32.59)	567 (57.74)	279 (55.36)
≥ 6	64 (5.45)	76 (6.53)	124 (12.63)	32 (6.35)
**Meat (times/week)**				
Never or < 7	29 (2.47)	152 (13.07)	134 (13.65)	71 (14.09)
7–14	483 (41.11)	486 (41.79)	497 (50.61)	255 (50.60)
14 or more	663 (56.43)	525 (45.14)	351 (35.74)	178 (35.32)
**Vegetable (times/week)**				
Never or < 7	24 (1.96)	35 (2.75)	88 (8.55)	29 (5.16)
7–14	434 (36.94)	521 (44.80)	499 (50.81)	252 (50.00)
14	717 (61.02)	607 (52.19)	395 (40.22)	223 (44.25)
**Fruit (times/week)**				
Never	15 (1.28)	221 (19.00)	93 (9.47)	64 (12.70)
< 7	572 (48.68)	724 (62.25)	754 (76.78)	367 (78.82)
7–14	556 (47.32)	203 (17.45)	123 (12.53)	64 (12.70)
14 or more	32 (2.72)	15 (1.29)	12 (1.22)	9 (1.79)
**Sweet(times/week)**				
Never	278 (23.66)	760 (65.35)	364 (37.07)	300 (59.52)
< 3	715 (60.85)	274 (23.56)	522 (53.16)	173 (34.33)
3–7	162 (13.79)	87 (7.48)	78 (7.94)	23 (4.56)
7 or more	20 (1.70)	42 (3.61)	18 (1.83)	8 (1.59)
**Exercise**				
No	330 (28.09)	614 (52.79)	524 (53.36)	280 (55.56)
Yes	845 (71.91)	549 (47.21)	458 (46.64)	224 (44.44)
**Sleep(hours/day)**				
< 5 or need medication	40 (3.40)	130 (11.18)	199 (20.26)	106 (21.03)
5–7	594 (50.55)	614 (52.79)	435 (44.30)	217 (43.06)
7–9	523 (44.51)	402 (34.57)	270 (27.49)	152 (30.16)
≥ 9	18 (1.53)	17 (1.46)	78 (7.94)	29 (5.75)
**BMI(kg/m)**^**2**^	23.43 ± 3.40	23.66 ± 3.98	22.99 ± 3.21	23.60 ± 3.28
**Central obesity**				
No	897 (76.09)	719 (61.82)	646 (65.78)	287 (56.94)
Yes	281 (23.91)	444 (38.18)	336 (34.22)	217 (43.06)
**SBP (mmHg)**	122.70 ± 16.11	135.79 ± 21.85	139.34 ± 21.21	142.15 ± 22.04
**DBP (mmHg)**	74.05 ± 11.05	77.52 ± 12.06	77.99 ± 12.10	77.17 ± 12.06
**Heart rate (times/min)**	79.49 ± 12.15	81.44 ± 12.18	74.12 ± 12.44	6.55 ± 11.64
**FBG (mmol/l)**	4.87(4.56,5.22)	7.80(5.64,1.40)	5.03(4.57,5.70)	7.61(5.47, .73)
**TG (mmol/l)**	1.13(0.81, .72)	1.47(1.00, .21)	1.29(0.94, .81)	1.48(1.04, .02)
**TC (mmol/l)**	5.20 ± 1.10	5.16 ± 1.47	4.81 ± 1.31	4.63 ± 1.35
**HDL-C (mmol/l)**	1.37 ± 0.36	1.21 ± 0.47	1.21 ± 0.33	1.14 ± 0.31
**LDL-C (mmol/l)**	2.67 ± 1.36	3.14 ± 1.28	2.89 ± 1.12	2.68 ± 1.08

Measurement data are expressed as$\overline{{\text{x}}}\pm \mathrm{s }$或*M(P25, P75)*, and counting data are expressed as n (%)

*BMI* body mass index, *SBP* Systolic blood pressure, *DBP* Diastolic blood pressure, *FBG* Fasting blood glucose, *TG* Triglyceride, *TC* Total cholesterol, *HDL-C *High density lipoprotein cholesterol, *LDL-C* Low density lipoprotein cholesterol

### Preliminary screening of influencing factors of type 2 diabetes, coronary heart disease and their comorbidity

Univariate analysis showed that for T2DM, except BMI and TC, there were differences in the distribution of other 24 variables between groups, and the differences were statistically significant (*P* < 0.05).For CAD, except gender and FHx of coronary heart disease, there were statistical differences in the distribution of other 25 variables between groups (*P* < 0.05).Univariate analysis of comorbidities showed that except gender, FHx of DM, FHx of CAD, BMI and LDL-C, there were statistically significant differences in the distribution of other 22 variables between groups (*P* < 0.05).The results of single factor analysis for preliminary screening of influencing factors of disease are shown in Table 2.

**Table 2:** Results of single factor analysis of disease influencing factors screening

Variable	T2DM		CHD		Comorbidity	
	*t/χ*^*2*^	*P*	*t/χ*^*2*^	*P*	*t/χ*^*2*^	*P*
**Age**	-35.502	< 0.001	-47.784	< 0.001	-43.520	< 0.001
**Sex**	12.411	< 0.001	2.803	0.094	0.002	0.964
**Area**	648.232	< 0.001	546.043	< 0.001	377.617	< 0.001
**Education**	1026.959	< 0.001	1056.097	< 0.001	694.446	< 0.001
**Marriage**	203.301	< 0.001	224.917	< 0.001	114.925	< 0.001
**Occupation**	901.096	< 0.001	809.569	< 0.001	545.586	< 0.001
**FHx of DM**	17.253	< 0.001	54.027	< 0.001	0.033	0.857
**FHx of CAD**	15.767	< 0.001	0.886	0.347	0.049	0.826
**Smoke**	33.334	< 0.001	72.664	< 0.001	28.791	< 0.001
**Drink**	128.925	< 0.001	127.989	< 0.001	88.035	< 0.001
**Staple food**	20.469	< 0.001	128.673	< 0.001	30.639	< 0.001
**Meat (times/week)**	99.566	< 0.001	147.752	< 0.001	118.550	< 0.001
**Vegetable (times/week)**	19.054	< 0.001	118.017	< 0.001	47.853	< 0.001
**Fruit (times/week)**	367.913	< 0.001	352.080	< 0.001	250.290	< 0.001
**Sweet (times/week)**	450.810	< 0.001	54.304	< 0.001	205.969	< 0.001
**Exercise**	148.235	< 0.001	142.887	< 0.001	115.065	< 0.001
**Sleep**	63.775	< 0.001	233.162	< 0.001	170.673	< 0.001
**BMI (kg/m)**^**2**^	-1.532	< 0.126^a^	3.085	0.002^a^	-0.963	0.336^a^
**Central obesity**	55.573	< 0.001	27.794	< 0.001	61.938	< 0.001
**SBP (mmHg)**	-16.477	< 0.001^a^	-20.191	< 0.001^a^	-17.864	< 0.001^a^
**DBP (mmHg)**	-7.250	< 0.001^a^	-7.845	< 0.001^a^	-4.989	< 0.001^a^
**Heart rate (times/min)**	-3.880	< 0.001^a^	10.118	< 0.001^a^	4.609	< 0.001^a^
**FBG (mmol/l)**	-	-	-5.257	< 0.001^b^	-20.689	< 0.001^b^
**TG (mmol/l)**	-10.037	< 0.001^b^	-5.262	< 0.001^b^	-8.471	< 0.001^b^
**TC (mmol/l)**	0.876	0.381^a^	7.460	< 0.001^a^	8.421	< 0.001^a^
**HDL-C (mmol/l)**	9.421	< 0.001^a^	11.019	< 0.001^a^	13.512	< 0.001^a^
**LDL-C (mmol/l)**	-8.548	< 0.001^a^	4.103	< 0.001^a^	-0.203	0.840^a^

Since fasting blood glucose is a diagnostic criterion for diabetes, it was not included in the analysis

^a^The t value of the statistic

^b^the Z value of the statistic, and the rest of the statistic are the *χ*^*2*^

### Logistic regression analysis of type 2 diabetes, coronary heart disease and their comorbidities

The more nodes (variables), the larger the sample size required to construct a reasonable BN, and too many network nodes are not conducive to reflecting the relationship between the main factors and the outcome. Therefore, variables with *P* < 0.05 in the univariate analysis were selected by logistic stepwise regression to simplify the structure of the later BN. The variables and their assignments are described in Table 3.

**Table 3:** Variables and their assignments

Variable	Assign
Sex	Male = 1^a^, female = 2
Area	Rural = 1^a^, suburban = 2, urban = 3
Education	Illiteracy (low) = 1^a^, primary, middle, high school or technical secondary school (middle) = 2, college, bachelor, master's degree and above (high) = 3
Marriage	Married = 1^a^, unmarried, divorced, widowed = 2
Occupation	Agriculture and forestry = 1^a^, business service = 2, military, national party = 3, students = 4, others = 5
FHx of DM	No = 0^a^, and Yes = 1
FHx of CAD	No = 0^a^, and Yes = 1
Smoke	Never = 1^a^, occasionally (< 10 cigarettes/day) = 2, often (≥ 10 cigarettes/day) = 3, quitting (no smoking in the last 6 months) = 4
Drink	Never = 1^a^, occasionally (< 3 times/week) = 2, often (≥ 3 times/week) = 3, quitting (no alcohol in the last 6 months) = 4
Staple food	Never or < 3 liang/day = 1^a^, 3–6 liang /day = 2, ≥ 6 liang /day = 3
Meat	Never or < 7 times/week = 1^a^, 7–14 times/week = 2,14 times or more/week = 3
Vegetable	Never or < 7 times/week = 1^a^, 7–14 times/week = 2,14 times or more/week = 3
Fruit intake	Never = 1^a^, < 7 times/week = 2, 7–14 times/week = 3,14 times or more/week = 4
Sweet	Never = 1^a^, < 3 times/week = 2, 3–7 times/week = 3, 7 times or more/week = 4
Exercise	No = 0^a^, and Yes = 1
Sleep	< 5 h/day or need medicine to help sleep = 1^a^, 5–7 h/day = 2, 7–9 h/day = 3, ≥ 9 h/day = 4

Table ^a^ indicates control level

Logistic regression results of T2DM showed that there were 16 factors entering the model, which were age, region, education level, occupation, FHx of DM smoking, drinking, intake of staple food, meat, fruit, sweets, exercise, heart rate, TG, HDL-C and LDL-C. Among them, the older the age, the lower the education level, the FHx of DM, the frequent smoking, the higher the risk of T2DM; Compared with suburbs and cities, the risk of disease in rural population was 1.798(1/0.556) times and 14.386 (1/0.070) times, respectively. Business service workers had a higher risk than other occupational groups. Occasional drinkers had a 58.9 percent lower risk than non-drinkers. The risk of disease was 2.299(1/0.435) times of those who did not eat staple foods or consumed less than 3 liang per day compared with 3–6 liang per day. Higher intake of meat, fruit and sweets was associated with a lower risk of T2DM; For each unit increase in heart rate, TG, LDL-C, and HDL-C, the likelihood of T2DM increased by 2.7%, 8.9%, 34.3%, and 62.3%, respectively. See Table 4 for details.

**Table 4:** Results of multivariate logistic regression analysis of T2DM

Variable	*Beta*	$\boldsymbol{S}_{\boldsymbol{\bar{x}}}$	Wald* χ*^2^	*P*	*OR*(95%*CI*)
**Age**	0.077	0.007	115.551	< 0.001	1.080(1.065 ~ 1.095)
**Area**					
Rural			124.932	< 0.001	
Suburban	-0.587	0.360	2.668	0.102	0.556(0.275 ~ 1.125)
Urban	-2.661	0.283	88.421	< 0.001	0.070(0.040 ~ 0.122)
**Education**					
Low			51.399	< 0.001	
Middle	0.070	0.511	0.019	0.892	1.072 0.393 ~ 2.922)
High	-1.290	0.537	5.775	0.016	0.275 0.096 ~ 0.788)
**Occupation**					
Agriculture and forestry			76.512	< 0.001	
Business services	1.243	0.331	14.082	< 0.001	3.467(1.811 ~ 6.638)
Military, national party	1.037	0.273	14.408	< 0.001	0.354(0.207 ~ 0.606)
Students	1.472	0.758	3.766	0.052	0.230(0.052 ~ 1.015)
Others	0.087	0.255	0.116	0.734	1.091 0.661 ~ 1.799)
**FHx of DM**	1.617	0.226	51.136	< 0.001	5.040 (3.235 ~ 7.852)
**Smoke**					
Never			14.581	0.002	
Occasionally	0.396	0.317	1.562	0.211	0.673 (0.361 ~ 1.253)
Often	0.667	0.235	8.092	0.004	1.949 (1.231 ~ 3.087)
Quitting	0.669	0.445	2.258	0.133	0.512 (0.214 ~ 1.226)
**Drink**					
Never			20.991	< 0.001	
Occasionally	-0.890	0.211	17.762	< 0.001	0.411 (0.271 ~ 0.621)
Often	-0.449	0.386	1.352	0.245	0.638 (0.300 ~ 1.360)
Quitting	0.562	0.501	1.260	0.262	1.754 (0.658 ~ 4.679)
**Staple food (liang/day)**					
Never or < 3			28.219	< 0.001	
3–6	0.833	0.177	22.286	< 0.001	0.435 (0.307 ~ 0.614)
≥ 6	0.344	0.324	1.127	0.288	1.411 (0.747 ~ 2.666)
**Meat (times/week)**					
Never or < 7			14.344	0.001	
7–14	1.112	0.381	8.504	0.004	0.329 (0.156 ~ 0.694)
14 or more	1.393	0.379	13.517	< 0.001	0.248 (0.118 ~ 0.522)
**Fruit (times/week)**					
Never			28.481	< 0.001	
< 7	1.551	0.403	14.825	< 0.001	0.212 (0.096 ~ 0.467)
7–14	2.100	0.417	25.295	< 0.001	0.122 (0.054 ~ 0.278)
14 or more	1.478	0.637	5.384	0.020	0.228 (0.065 ~ 0.795)
**Sweet (times/week)**					
Never			40.793	< 0.001	
< 3	1.058	0.180	34.591	< 0.001	0.347 (0.244 ~ 0.494)
3–7	0.535	0.269	3.951	0.047	0.586 (0.346 ~ 0.993)
7 or more	0.516	0.455	1.287	0.257	1.675 (0.687 ~ 4.082)
**Exercise**	0.920	0.166	30.823	< 0.001	0.399 (0.288 ~ 0.552)
**Heart rate (times/min)**	0.027	0.007	15.924	< 0.001	1.027 (1.014 ~ 1.041)
**TG (mmol/l)**	0.085	0.041	4.272	0.039	1.089 (1.004 ~ 1.180)
**HDL-C (mmol/l)**	0.975	0.170	32.816	< 0.001	0.377 (0.270 ~ 0.527)
**LDL-C (mmol/l)**	0.417	0.062	44.921	< 0.001	1.343 (1.340 ~ 1.714)

The logistic regression results of CAD showed that there were 17 factors that finally entered the regression model, which were age, region, education level, marital status, occupation, smoking, drinking, staple food, meat, fruit intake, exercise, sleep time, SBP, heart rate, TG, TC and LDL-C. The risk of CAD increased by 0.091 times, 0.020 times, 0.140 times and 2.197 times for each unit increase of age, SBP, TG and LDL-C, respectively. People who live in rural areas, have low education level, often smoke, eat more staple food and do not exercise have an increased risk of CAD. Compared with married people, unmarried, divorced and widowed people have a lower risk of CAD. The risk of disease among military/national party and government personnel is lower than that of other occupational groups; Occasional drinkers had a 48.1% lower risk than non-drinkers. Compared with those who slept less than 5 h/day or needed medication to help them sleep, the risk of 5–7 h/day and 7–9 h/day were reduced by 51.8% and 48.6%, respectively. Higher meat and fruit intake was also associated with a lower risk of CAD. See Table 5 for details.

**Table 5:** Results of multivariate logistic regression analysis of CAD

Variable	*Beta*	$\boldsymbol{S}_{\boldsymbol{\bar{x}}}$	Wald* χ*^2^	* P*	* OR (95% CI )*
**Age**	0.087	0.008	112.342	< 0.001	1.091(1.074 ~ 1.109)
**Area**					
Rural			40.217	< 0.001	
Suburban	1.486	0.427	12.110	0.001	0.226(0.098 ~ 0.523)
Urban	2.067	0.329	39.407	< 0.001	0.127(0.066 ~ 0.241)
**Education**					
Low			30.364	< 0.001	
Middle	0.373	0.671	0.309	0.578	0.689(0.185 ~ 2.564)
High	1.553	0.695	4.993	0.025	0.212(0.054 ~ 0.826)
**Marriage**	1.299	0.428	9.208	0.002	0.273(0.118 ~ 0.631)
**Occupation**					
Agriculture and forestry			24.161	< 0.001	
Business services	0.284	0.409	0.482	0.488	1.328 (0.596 ~ 2.957)
Military, national party	0.957	0.298	10.345	0.001	0.384 (0.214 ~ 0.688)
Students	2.146	2.001	1.150	0.283	0.117 (0.002 ~ 5.902)
Others	0.006	0.293	0.000	0.985	0.994 (0.560 ~ 1.764)
**Smoke**					
Never			20.055	< 0.001	
Occasionally	0.387	0.330	1.373	0.241	1.472 (0.771 ~ 2.812)
Often	1.174	0.264	19.828	< 0.001	3.235 (1.929 ~ 5.423)
Quitting	0.144	0.436	0.110	0.740	1.155 (0.492 ~ 2.713)
**Drink**					
Never			7.898	0.048	
Occasionally	0.656	0.238	7.580	0.006	0.519 (0.326 ~ 0.828)
Often	0.013	0.373	0.001	0.972	0.987 (0.475 ~ 2.052)
Quitting	0.288	0.679	0.180	0.671	0.750 (0.198 ~ 2.835)
**Staple food (liang/day)**					
Never or < 3			13.188	0.001	
3–6	0.420	0.206	4.135	0.042	1.521 (1.015 ~ 2.280)
≥ 6	1.198	0.335	12.797	< 0.001	3.314 (1.719 ~ 6.388)
**Meat (times/week)**					
Never or < 7			6.668	0.036	
7–14	1.116	0.433	6.649	0.010	0.328 (0.140 ~ 0.765)
14 or more	1.033	0.436	5.608	0.018	0.356 (0.151 ~ 0.837)
**Fruit (times/week)**					
Never			25.911	< 0.001	
< 7	1.461	0.559	6.838	0.009	0.232 (0.078 ~ 0.694)
7–14	2.339	0.580	16.262	< 0.001	0.096 (0.031 ~ 0.301)
14 or more	1.845	0.780	5.602	0.018	0.158 (0.034 ~ 0.728)
**Exercise**	0.518	0.192	7.263	0.007	0.596 (0.409 ~ 0.868)
**Sleep**					
< 5 or need medication			13.365	0.004	
5–7	0.730	0.316	5.316	0.021	0.482 (0.259 ~ 0.896)
7–9	0.666	0.327	4.137	0.042	0.514 (0.271 ~ 0.976)
≥ 9	0.934	0.626	2.230	0.135	2.546 (0.747 ~ 8.679)
**SBP (mmHg)**	0.019	0.005	14.343	< 0.001	1.020 (1.009 ~ 1.030)
**Heart rate (times/min)**	0.023	0.008	9.076	0.003	0.978 (0.963 ~ 0.992)
**TG (mmol/l)**	0.131	0.062	4.450	0.035	1.140 (1.009 ~ 1.287)
**TC (mmol/l)**	1.271	0.131	94.262	< 0.001	0.280 (0.217 ~ 0.363)
**LDL-C (mmol/l)**	1.162	0.125	86.172	< 0.001	3.197 (2.502 ~ 4.087)

alogistic regression results of comorbidities showed that age, region, education level, fruit, sweet food intake, exercise, SBP, DBP, FBG, TC and HDL-C were related to 11 variables. The risk of comorbidities increased by 10.6%, 2.6%, 2.9% and 49.6% for each grade of age, systolic blood pressure, diastolic blood pressure and FBG.With each increase of TC and HDL-C grade, the risk of disease was reduced by 32.3% and 85% respectively. The risk of comorbidity in rural areas was 9.091(1/0.110) times that in urban areas. People with a college degree or above were 82% less likely to get the disease than those who were illiterate. Compared with no fruit, the probability of comorbidities decreased when the intake of fruit was 7–14 times or less per week. Those who exercised had a 49.3 percent lower risk than those who did not. See Table 6 for details.

**Table 6:** Results of multivariate logistic regression analysis of comorbidity

Variable	*Beta*	$\boldsymbol{S}_{\boldsymbol{\bar{x}}}$	Wald* χ*^2^	*P*	*OR*(95%*CI*)
**Age**	0.100	0.010	98.677	< 0.001	1.106 (1.084 ~ 1.128)
**Area**					
Rural			50.027	< 0.001	
Suburban	0.570	0.471	1.462	0.227	0.565(0.22 ~ 1.425)
Urban	2.207	0.370	35.589	< 0.001	0.110 (0.053 ~ 0.227)
**Education**					
Low			34.771	< 0.001	
Middle	0.158	0.662	0.057	0.812	0.854 (0.233 ~ 3.127)
High	1.716	0.692	6.149	0.013	0.180 (0.046 ~ 0.698)
**Fruit (times/week)**					
Never			27.138	< 0.001	
< 7	1.423	0.610	5.446	0.020	0.241 (0.073 ~ 0.796)
7–14	2.544	0.639	15.840	< 0.001	0.079 (0.022 ~ 0.275)
14 or more	0.977	0.888	1.211	0.271	0.376 (0.066 ~ 2.144)
**Sweet (times/week)**					
Never			9.463	0.024	
< 3	0.704	0.231	9.260	0.002	0.495(0.314 ~ 0.778)
3–7	0.498	0.406	1.498	0.221	0.608 (0.274 ~ 1.349)
7 or more	0.209	0.672	0.096	0.756	0.812 (0.217 ~ 3.032)
**Exercise**	0.680	0.228	8.908	0.003	0.507 (0.324 ~ 0.792)
**SBP (mmHg)**	0.026	0.008	11.120	0.001	1.026 (1.011 ~ 1.042)
**DBP (mmHg)**	-0.028	0.013	4.837	0.028	0.972 (0.948 ~ 0.997)
**FBG (mmol/l)**	0.403	0.059	46.775	< 0.001	1.496 (1.333 ~ 1.679)
**TC (mmol/l)**	0.388	0.085	20.739	< 0.001	0.678 (0.574 ~ 0.802)
**HDL-C (mmol/l)**	1.895	0.363	27.194	< 0.001	0.150 (0.074 ~ 0.307)

### BN learning

#### Structure learning

Variables with statistically significant differences in multivariate Logistic regression analysis were selected, and 70% of T2DM, CAD and their comorbiditis data were randomly selected as the training set. Tabu search algorithm was used to learn the structure of BN, and the BN model was constructed by combining the prior knowledge of experts and data information. Structure learning needs to discretize continuous variables, which can not only improve the accuracy of network learning, but also reduce the risk of model overfitting, making the data mining results more practical value. Table 7 shows the variable-discretization rules.

**Table 7:** Variable discretization rules

Variable	Assign
Age	18 ~ 44 = 1, 45 ~ 59 = 2,60 ~ 3
BMI (kg/m)^2^	< 18.49 = 1,18.5 ~ 23.99 = 2,24.0 ~ 27.99 = 3,28.0 ~ = 4
SBP (mmHg)	Normal = 0 (< 140), abnormal = 1 (≥ 140)
DBP (mmHg)	Normal = 0 (< 90), abnormal = 1 (≥ 90)
Heart rate (times/min)	Too slow = 1 (< 60), normal = 2 (60 ~ 100), too fast = 3 (> 100)
FBG (mmol/L)	Normal = 0 (< 7.0), abnormal = 1 (≥ 7.0)
TG (mmol/L)	Normal = 0 (< 2.3), abnormal = 1 (≥ 2.3)
TC (mmol/L)	Normal = 0 (< 6.2), abnormal = 1 (≥ 6.2)
HDL-C (mmol/L)	Normal = 0 (< 2.3), abnormal = 1 (≥ 2.3)
LDL-C (mmol/L)	Normal = 0 (≥ 1.0), abnormal = 1 (< 1.0)

**Fig. 1:**
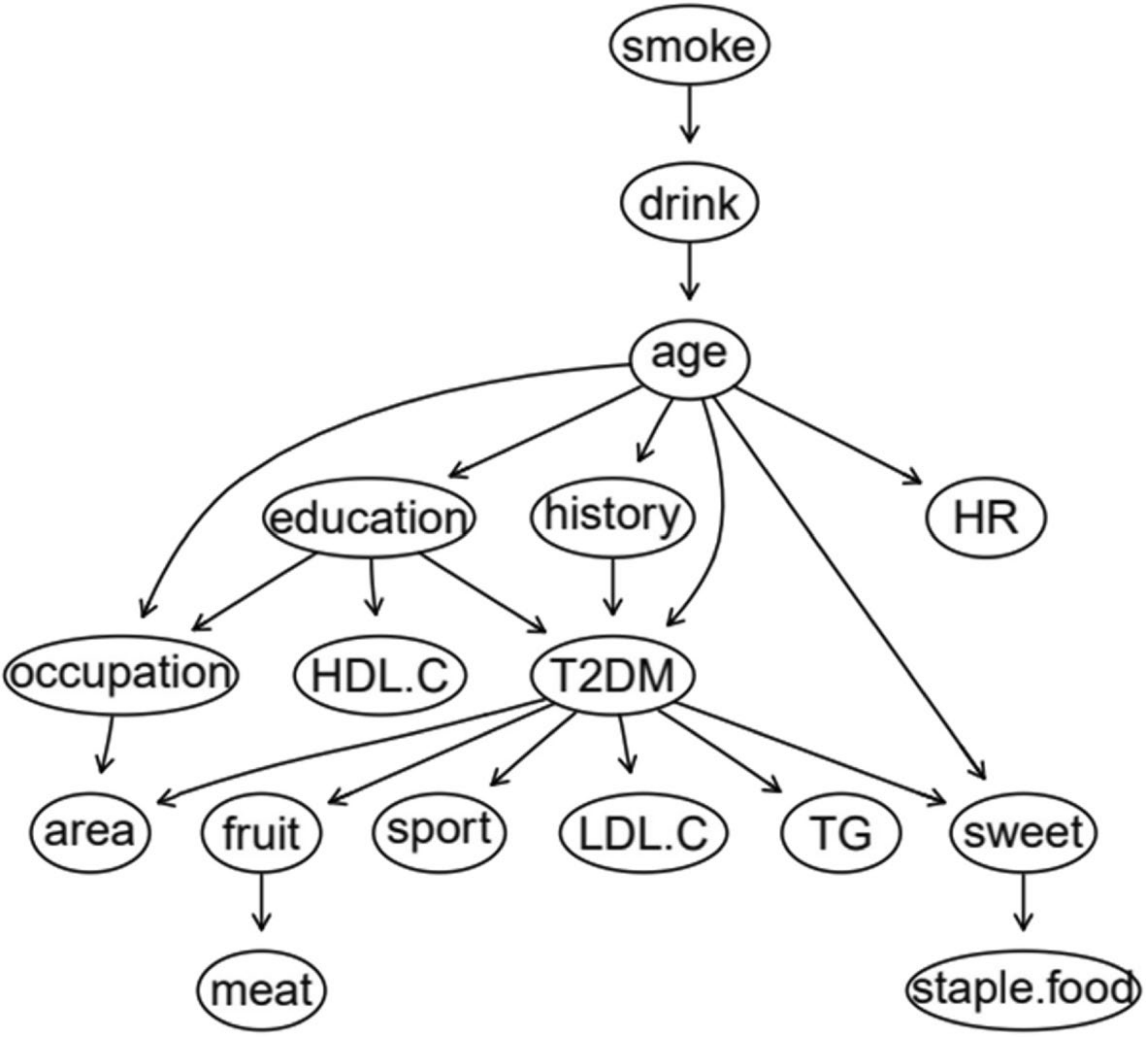
BN diagram of influencing factors of T2DM

The BN of T2DM influencing factors is shown in Fig. 1, which contains 17 nodes and 21 directed edges. The directed edge represents the dependent relationship between related factors and T2DM. The network structure shows that age, education level and FHx are the parent nodes of T2DM, that is, they are the direct influencing factors of T2DM. Smoking, alcohol consumption, heart rate, occupation, HDL-C, staple food and meat intake indirectly affect T2DM by influencing other factors. Region, fruit intake, exercise, LDL-C, TG and sweet food intake are the subnodes of T2DM, that is, they are also directly related to T2DM. Among them, age, region, education level, FHx and sweet food intake, in addition to the direct effect on T2DM, can also be indirectly related to other factors.

**Fig. 2:**
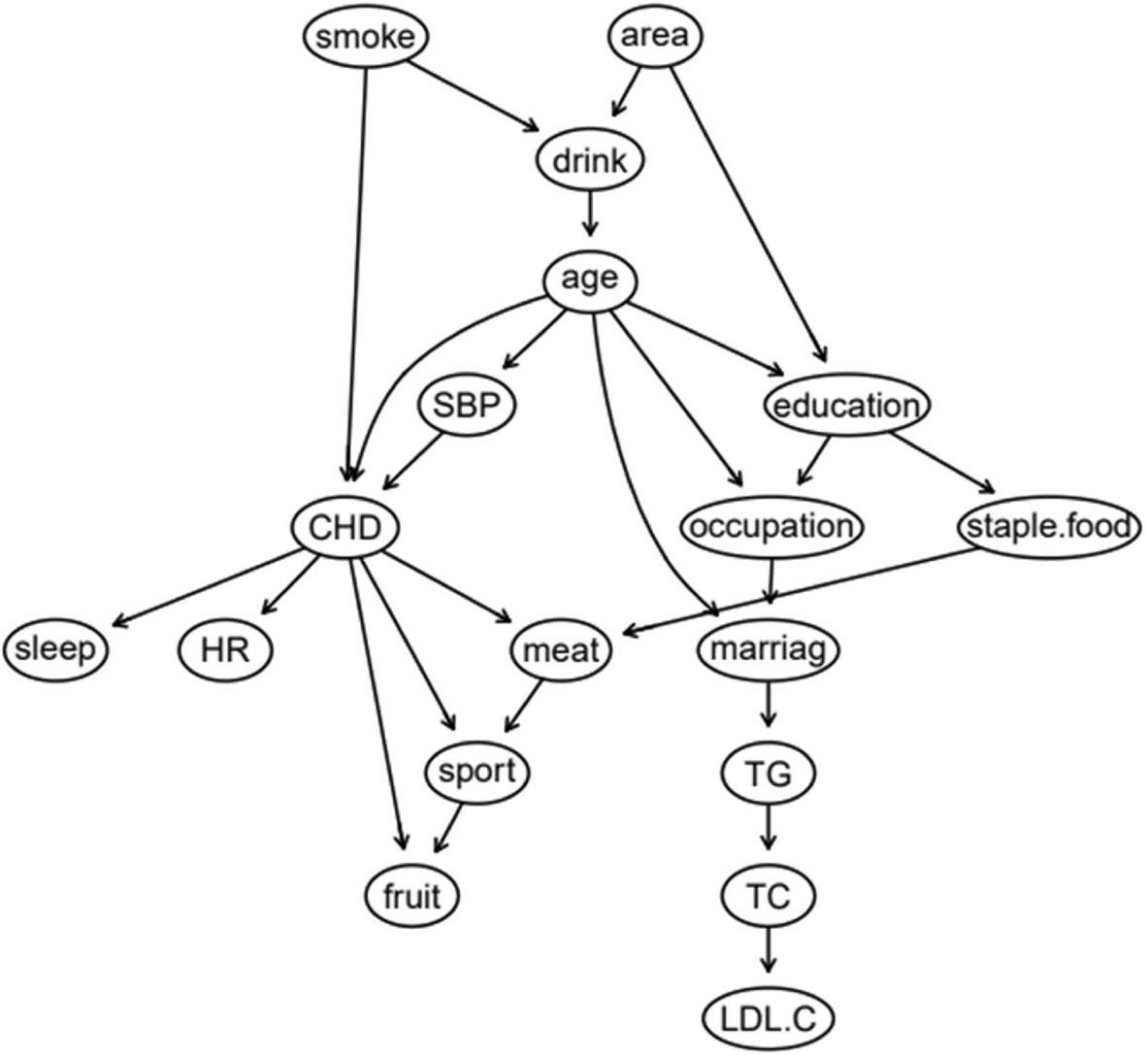
BN diagram of influencing factors of CAD

The BN of CAD influencing factors is shown in Fig. 2, which contains 18 nodes and 25 directed edges. The network structure showed that age, SBP, smoking, sleep time, heart rate, exercise, meat and fruit intake were directly related to CAD, Alcohol consumption, educational level, occupation and marital status can be indirectly correlated with CAD through other nodes, and other variables were correlated, but the network relationship with CAD was far away.

**Fig. 3:**
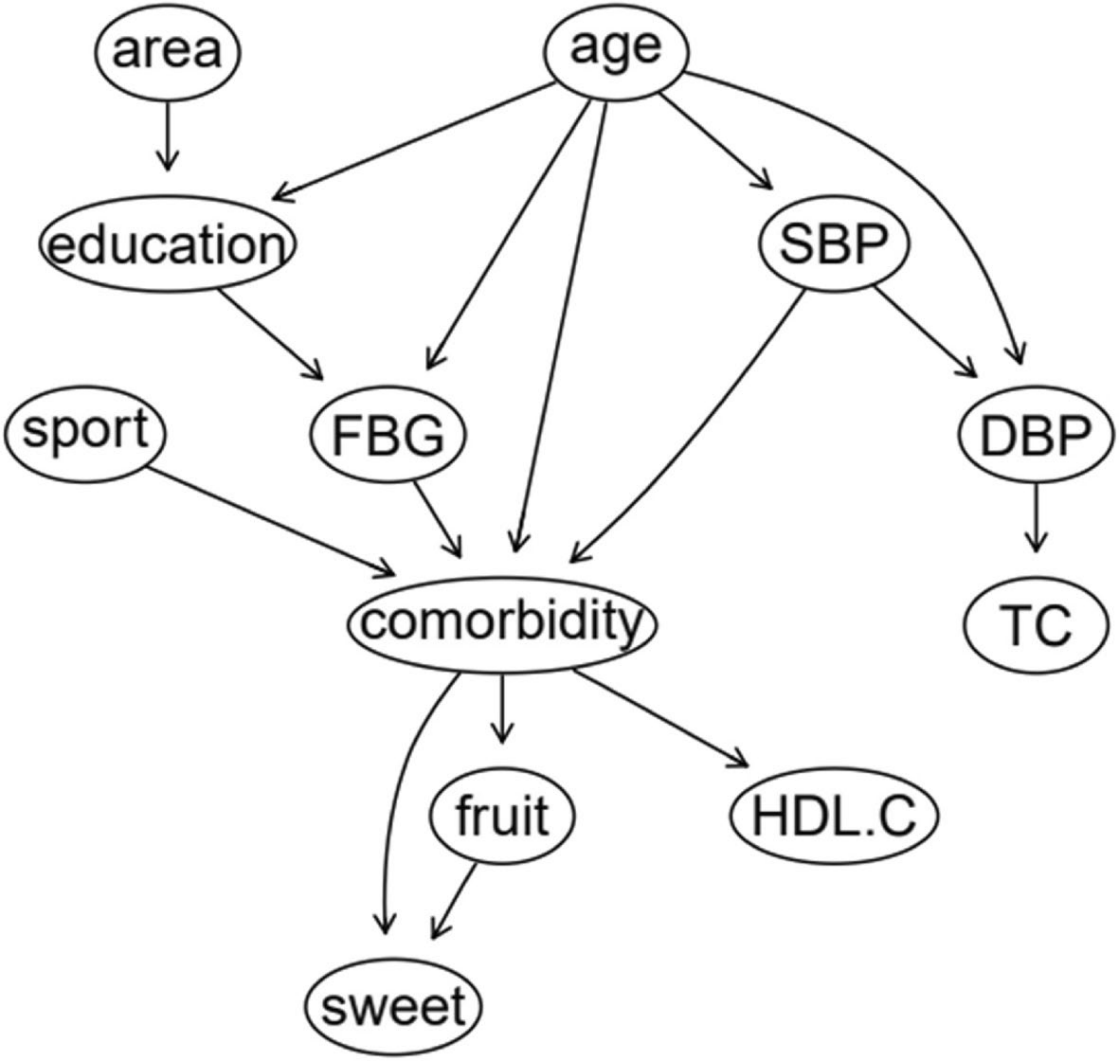
BN diagram of influencing factors of comorbidity

The BN of comorbidity influencing factors is shown in Fig. 3, which contains 12 nodes and 16 directed edges. The network structure showed that age, FBG, SBP, exercise, sweets, fruit intake and HDL-C were directly related to comorbidity. Among them, age and sweet food intake had both direct and indirect effects on comorbidities. Region and education level were indirectly associated with comorbidities through FBG, TC and DBP were indirectly associated with comorbidities through SBP.

#### Parameter learning

For the constructed BN structure, the maximum likelihood estimation method is used for parameter learning. Table 8 is the conditional probability table for T2DM as child node. It can be seen that the incidence probability of T2DM increases significantly with the increase of age. The incidence of T2DM decreased with higher education level. The incidence of DM in people with a FHx of DM is significantly higher than that in people without a FHx.

**Table 8:** Conditional probability of influencing factors of T2DM

Age	Education	FHx	T2DM
18 to 44	Low	No	0.500
45 ~ 59	Low	No	0.833
60 ~	Low	No	0.935
18 to 44	Middle	No	0.404
45 ~ 59	Middle	No	0.737
60 ~	Middle	No	0.840
18 to 44	High	No	0.032
45 ~ 59	High	No	0.198
60 ~	High	No	0.307
18 to 44	Low	Yes	0.000
45 ~ 59	Low	Yes	1.000
60 ~	Low	Yes	1.000
18 to 44	Middle	Yes	0.826
45 ~ 59	Middle	Yes	0.915
60 ~	Middle	Yes	0.981
18 to 44	High	Yes	0.094
45 ~ 59	High	Yes	0.500
60 ~	High	Yes	0.857

Table 9 is a conditional probability table with CAD as a child node. It can be seen that the incidence probability of CAD increases with age.The incidence rate of regular smokers is much higher than that of non-smokers, and this gap is especially obvious in middle-aged and elderly people. The incidence of CAD in patients with abnormal SBP is significantly higher than that in normal population.

**Table 9:** Conditional probability of CAD influencing factors

Age	Smoke	SBP	CAD
18 to 44	Never	normal	0.018
45 ~ 59	Never	normal	0.383
60 ~	Never	normal	0.774
18 to 44	Occasionally	normal	0.021
45 ~ 59	Occasionally	normal	0.400
60 ~	Occasionally	normal	0.619
18 to 44	Often	normal	0.235
45 ~ 59	Often	normal	0.556
60 ~	Often	normal	0.902
18 to 44	Quitting	normal	0.000
45 ~ 59	Quitting	normal	0.666
60 ~	Quitting	normal	0.824
18 to 44	Never	abnormal	0.040
45 ~ 59	Never	abnormal	0.482
60 ~	Never	abnormal	0.863
18 to 44	Occasionally	abnormal	0.000
45 ~ 59	Occasionally	abnormal	0.875
60 ~	Occasionally	abnormal	0.958
18 to 44	Often	abnormal	0.333
45 ~ 59	Often	abnormal	0.778
60 ~	Often	abnormal	0.933
18 to 44	Quitting	abnormal	0.000
45 ~ 59	Quitting	abnormal	0.750
60 ~	Quitting	abnormal	0.826

Table 10 is a conditional probability table for children with comorbidity. Similarly, the probability of comorbidity increases with age. The incidence rate of inactive people was higher than that of exercisers. The older the age, the more obvious the difference was. Abnormal SBP and FBG can significantly increase the incidence of comorbidity, and the effect of FBG is stronger than that of SBP.

**Table 10:** Conditional probability of factors influencing comorbidity

Age	Exercise	SBP	FBG	Comorbidity
18 to 44	No	normal	normal	0.000
45 ~ 59	No	normal	normal	0.326
60 ~	No	normal	normal	0.712
18 to 44	Yes	normal	normal	0.000
45 ~ 59	Yes	normal	normal	0.098
60 ~	Yes	normal	normal	0.436
18 to 44	No	abnormal	normal	0.000
45 ~ 59	No	abnormal	normal	0.368
60 ~	No	abnormal	normal	0.843
18 to 44	Yes	abnormal	normal	0.000
45 ~ 59	Yes	abnormal	normal	0.292
60 ~	Yes	abnormal	normal	0.543
18 to 44	No	normal	abnormal	0.667
45 ~ 59	No	normal	abnormal	0.722
60 ~	No	normal	abnormal	0.977
18 to 44	Yes	normal	abnormal	0.000
45 ~ 59	Yes	normal	abnormal	0.667
60 ~	Yes	normal	abnormal	0.864
18 to 44	No	abnormal	abnormal	0.000
45 ~ 59	No	abnormal	abnormal	0.909
60 ~	No	abnormal	abnormal	0.964
18 to 44	Yes	abnormal	abnormal	0.250
45 ~ 59	Yes	abnormal	abnormal	0.769
60 ~	Yes	abnormal	abnormal	0.900

#### Network authentication

**Fig. 4:**
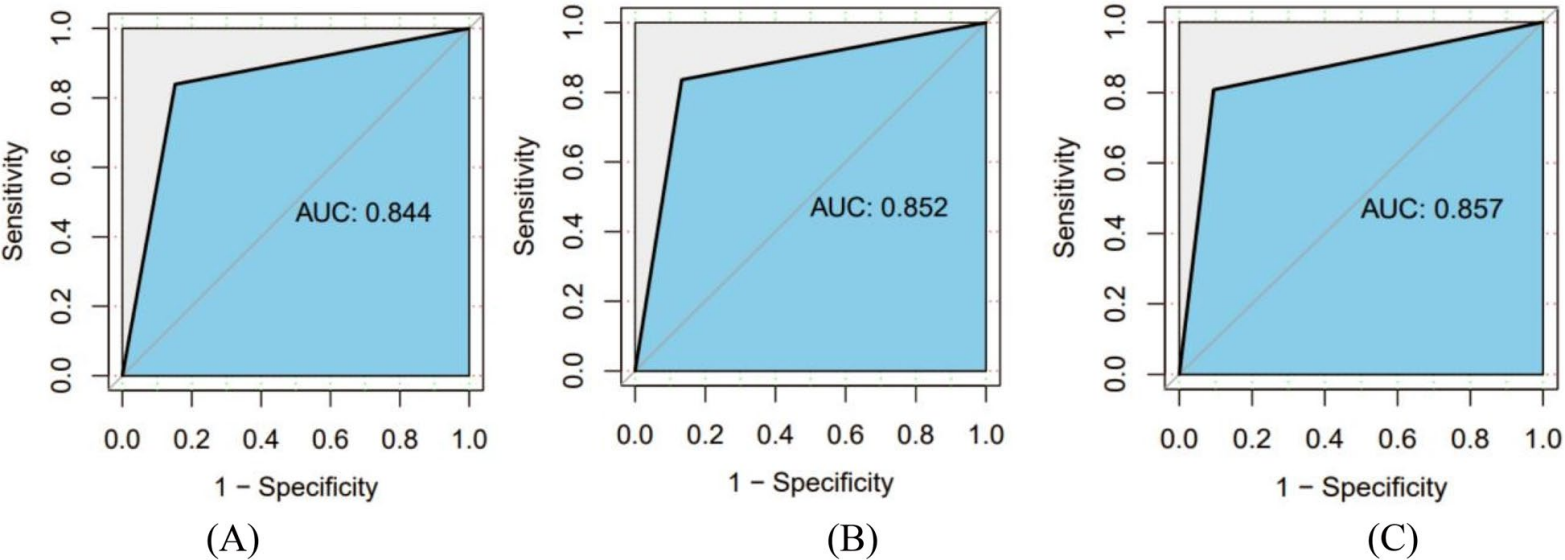
ROC curves of each disease model. Note: (**A**) ROC curve of T2DM prediction, (**B**) ROC curve of CAD prediction, (**C**) ROC curve of comorbidity prediction

After completing the BN structure learning and parameter learning, the remaining 30% data was taken as the test set, and the confusion matrix obtained was shown in Table 11. The results showed that the accuracy rate of T2DM prediction was 84.33%, the accuracy rate was 83.91%, the sensitivity was 86.23%, and the specificity was 82.30%. The area under ROC is 0.844 (95CI%:0.817 ~ 0.871) (Fig. 4A). The accuracy, accuracy, sensitivity and specificity of CAD prediction were 85.34%, 83.62%, 83.62%, 86.72%, and the area under ROC curve was 0.852 (95CI%:0.824 ~ 0.880) (Fig. 4B).The accuracy of comorbidity prediction was 87.62%, the accuracy was 80.79%, the sensitivity was 78.71%, the specificity was 91.62%, and the area under ROC curve was 0.857(95CI%:0.822 ~ 0.892) (Fig. 4C).The results of each evaluation index show that the three BN models have good prediction performance.

**Table 11:** Confusion matrix by disease

		T2DM		CAD		Comorbidity	
True value		**( +)**	**(-)**	**( +)**	**(-)**	**( +)**	**(-)**
Predicted value	**( +)**	313	60	240	47	122	29
	**(-)**	50	279	47	307	33	317

#### Network reasoning

BNs can use the conditional probability distribution determined by network structure and parameter learning to realize predictive reasoning and diagnostic reasoning of uncertain events, and can use third-party software to more intuitively display the complex relationship and probability distribution between variables and outcomes. Its biggest advantage is that it can automatically update the network probability by using Bayes theorem according to the different degree of information. In this study, the learning results are substituted into Netica, where the directed arc between nodes is expressed as the probability dependence between the connected nodes, and the nodes contain different states of each variable.

##### Predictive inference

**Fig. 5:**
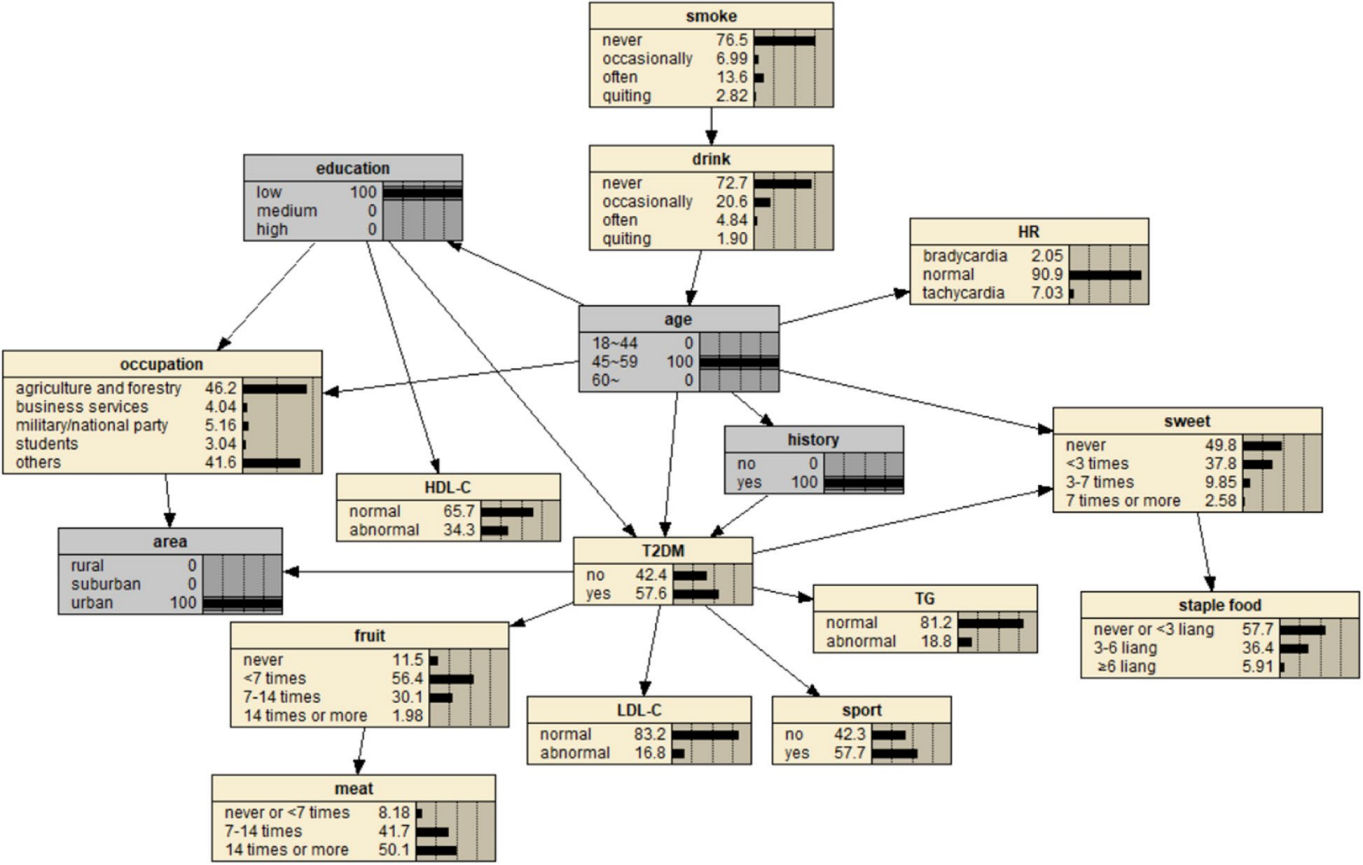
Prediction inference of T2DM influencing factors by BN model I

**Fig. 6:**
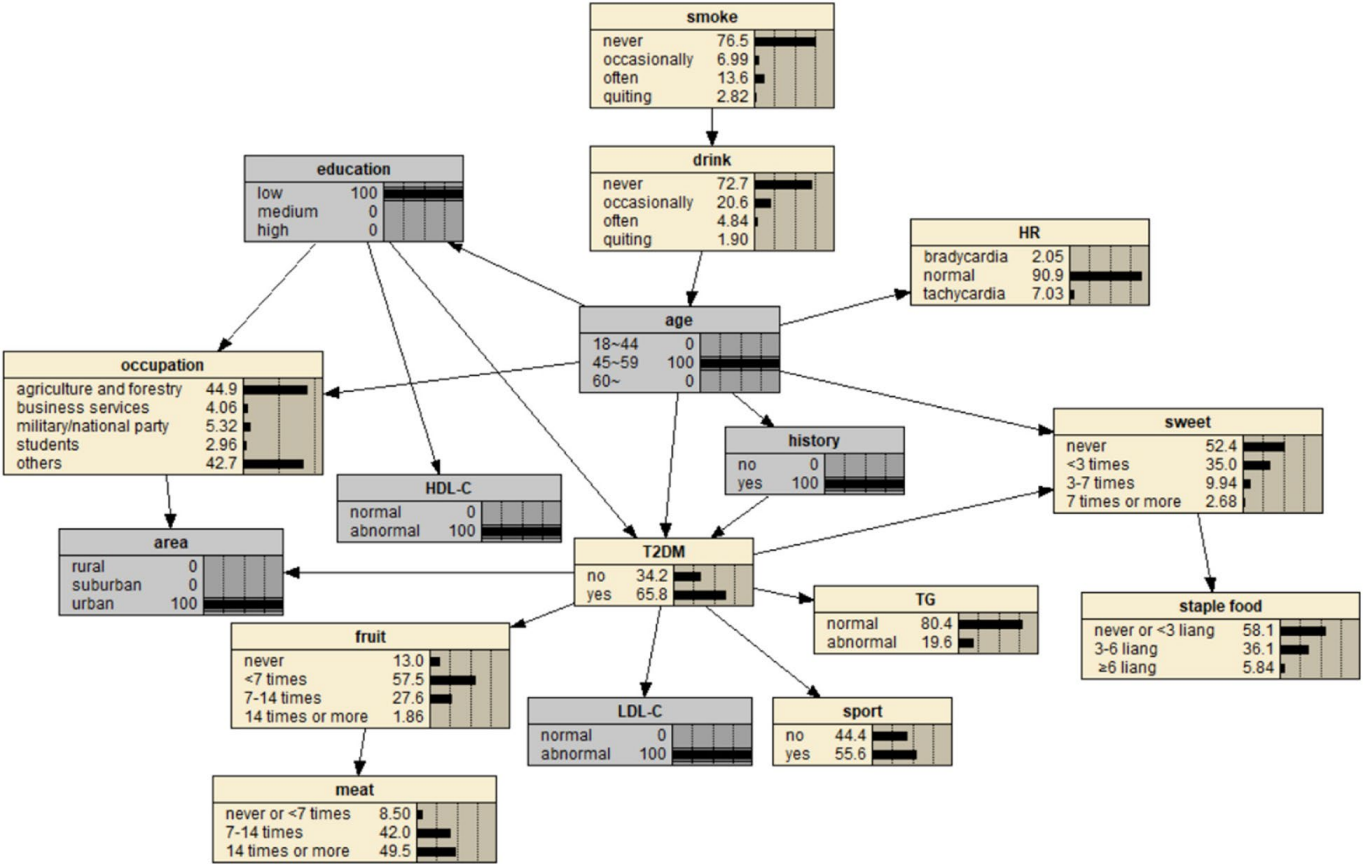
Prediction inference of T2DM influencing factors by BN model II

**Fig. 7:**
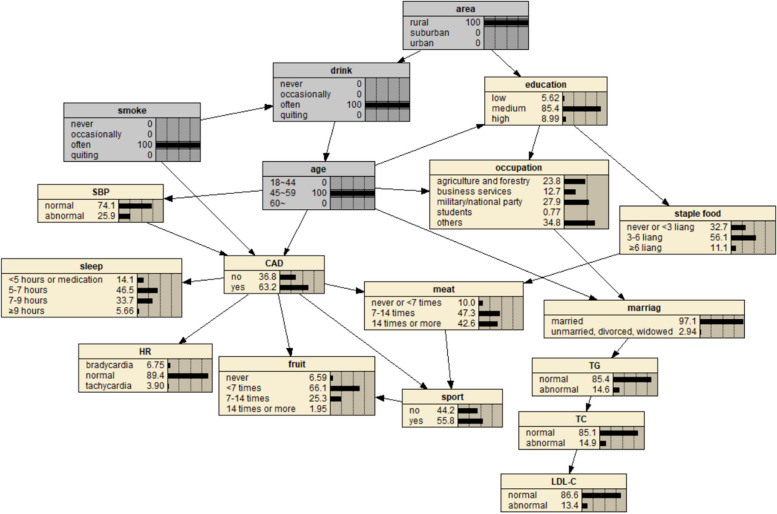
Prediction inference of CAD influencing factors by BN model I

**Fig. 8:**
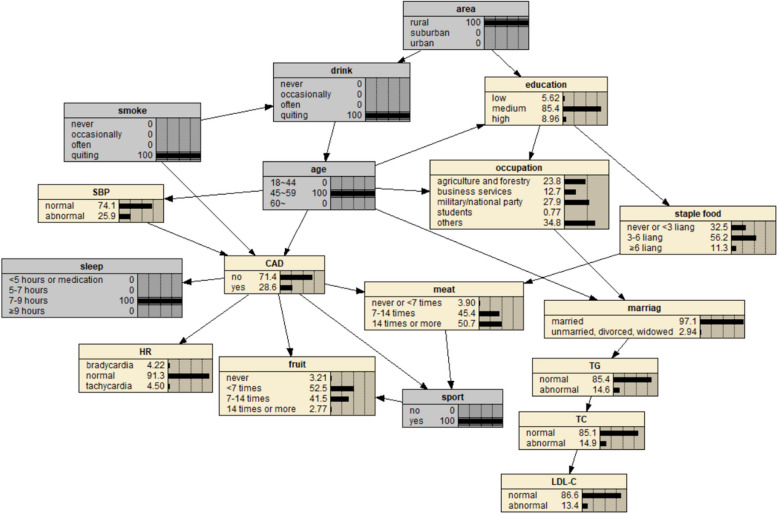
Prediction inference of CAD influencing factors by BN model II

**Fig. 9:**
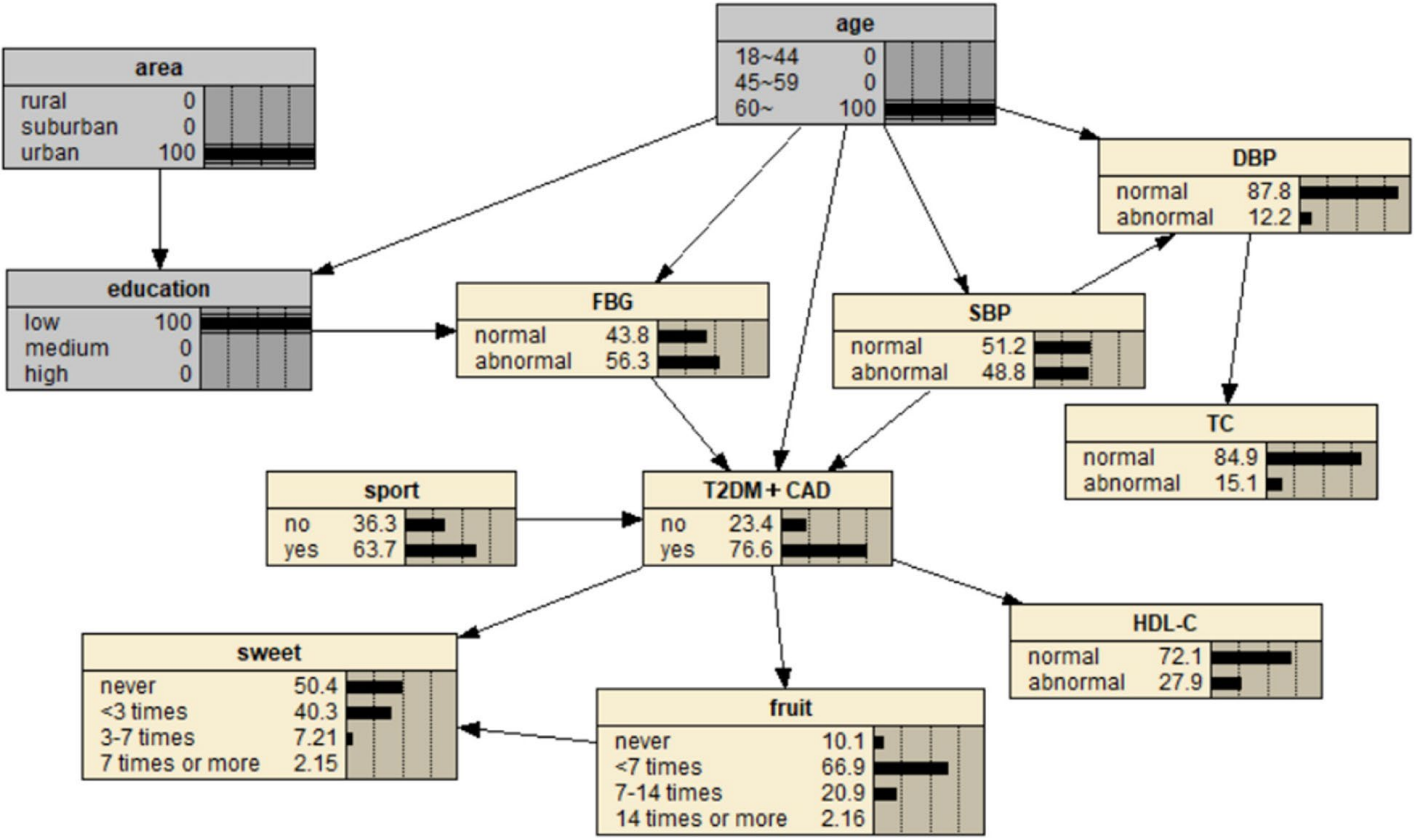
Prediction inference of comorbidity influencing factors by BN model I

**Fig. 10:**
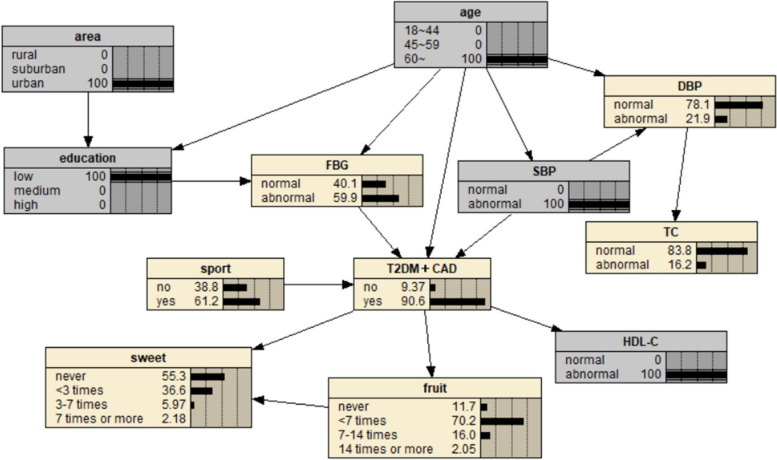
Prediction inference of comorbidity influencing factors by BN model II

Predictive reasoning is based on the prior probability of a specific basic event (node), and uses the conditional probability relationship between nodes to find out the probability of a node arising from the cause, that is, through some characteristics of known research objects, to predict the incidence probability of diseases. Suppose a study subject is known to be 45 to 59 years of age, illiterate, urban, and has a FHx of DM with a 57.6% risk of T2DM (Fig. 5). If the person is found to have abnormal HDL-C and LDL-C on clinical examination, the risk of T2DM is 57.6%.The BN showed that the risk of T2DM increased to 65.8% (Fig. 6), suggesting that patients with a FHx of DM and hyperlipidemia should be paid enough attention to reduce the risk of T2DM. If the subjects were aged 45–59 years old, lived in rural areas, and regularly smoked and drank alcohol, the risk of CAD was 63.2% (Fig. 7); if they quit smoking and drinking, and maintained exercise and adequate sleep, the risk of CAD was reduced to 28.6% (Fig. 8), indicating that a healthy lifestyle can effectively reduce the risk of coronary heart disease. If an individual is 60 years of age or older, illiterate, and living in an urban area, the risk of comorbidity is 76.6% (Fig. 9). If the person is found to have abnormal SBP and HDL-C by further biochemical examination, the risk of comorbidity increases to 90.6% (Fig. 10), indicating that hypertension and hyperlipidemia are closely related to comorbidity. Therefore, people with hypertension and hyperlipidemia, especially the elderly, should be given adequate attention to prevent T2DM and CAD.

##### Diagnostic reasoning

**Fig. 11:**
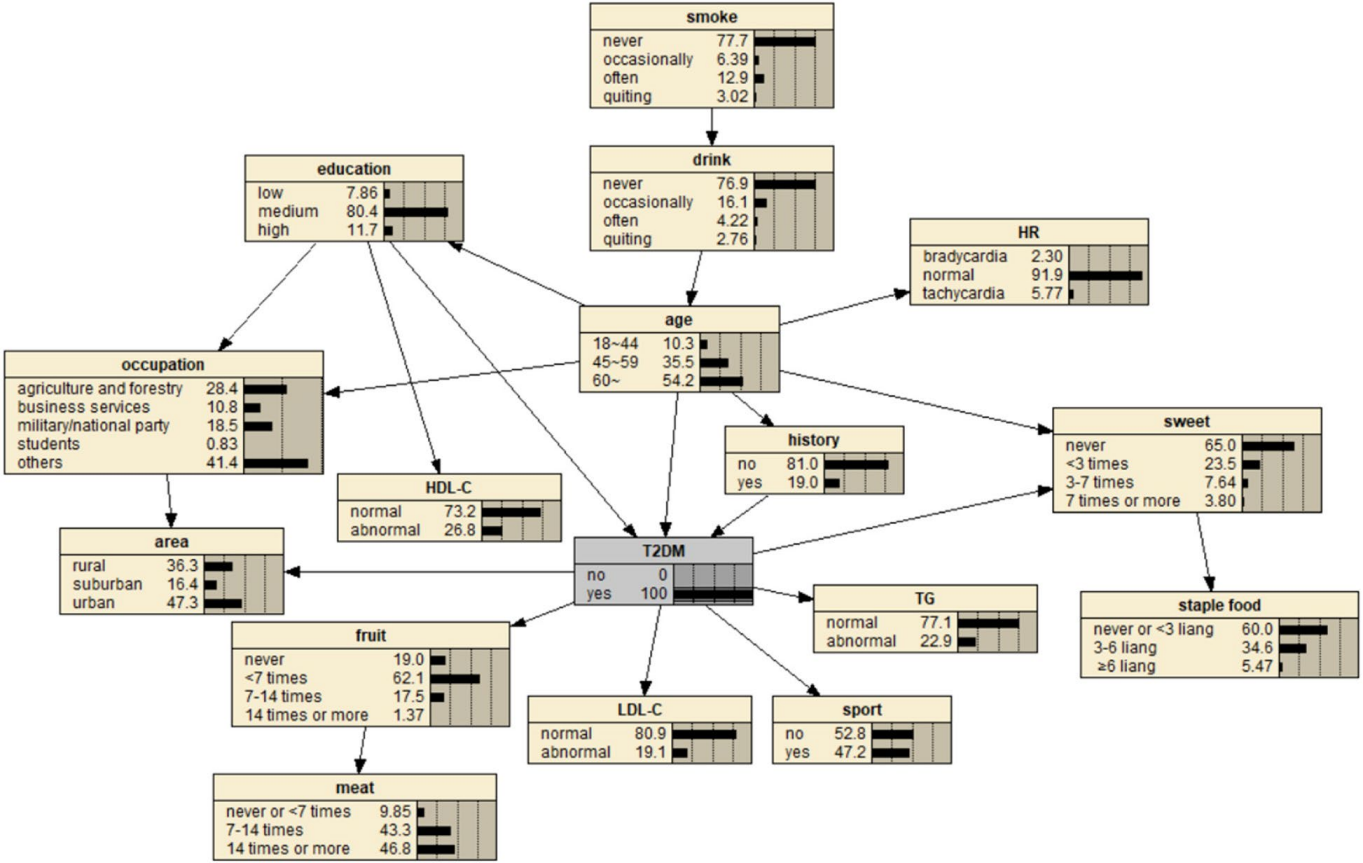
Diagnostic inference of BN model for T2DM influencing factors

BNs can not only predict the risk of outcome events, but also explore the conditional probability of causes through diagnostic reasoning, that is, on the premise of knowing the disease state, judge the basic situation of the inference research object and find out the pathogenic conditions. As shown in Fig. 11, after the incidence probability of T2DM is set to 100%, the change of node probability is observed. For example, in terms of age, the probability of people aged 18–44 years decreased from 38.1% to 10.3%, the probability of people aged 45–59 years increased from 29.1% to 35.5%, and the probability of people aged 60 years and above increased from 32.8% to 54.2%. The increase value was the largest, indicating that the elderly are the population with the highest risk of T2DM. For regions, the rural probability increased from 19.6% to 36.3%, and the suburban probability increased from 10.6% to 16.4%, indicating that people living in rural and suburban areas were more likely to get sick, and the rural population was more likely to get sick than the suburban population. In terms of education level, the probability of college and above decreased, and the probability of illiteracy and primary school, middle school, high school or secondary school increased, respectively, 7.86% and 80.4%, indicating that these two groups of people are more likely to suffer from T2DM. In terms of occupation, the probability value of agriculture and forestry personnel increased from 18.3% to 28.4%, and the probability value of business service personnel increased from 9.69% to 10.8%, indicating that these two occupational groups are more likely to get the disease, and the incidence rate of agriculture and forestry personnel may be higher than that of business service personnel. The probability of having a FHx increased from 16.3% to 19.0%, indicating that having a FHx increases the risk of T2DM. In terms of heart rate, the value of bradygia increased from 1.83% to 2.30%, the value of normal was unchanged, and the value of tachycardia decreased from 6.31% to 5.77%. The overall change was small, suggesting that bradygia may increase the incidence probability of T2DM, but the influence is small. In terms of dietary habits, the probability value of no eating or fruit intake less than 7 times/week, no eating or sweet food intake more than 7 times/week, meat intake 7–14 times/week or less, no eating or staple food intake less than 32 days increased, indicating a higher likelihood of T2DM. The probability of occasional drinking decreased, suggesting that moderate drinking may reduce the probability of the disease, while the probability of smoking did not change much. In addition, the probability values of no exercise and abnormal TG, HDL-C and LDL-C indexes also increased, indicating a greater probability of T2DM.

**Fig. 12:**
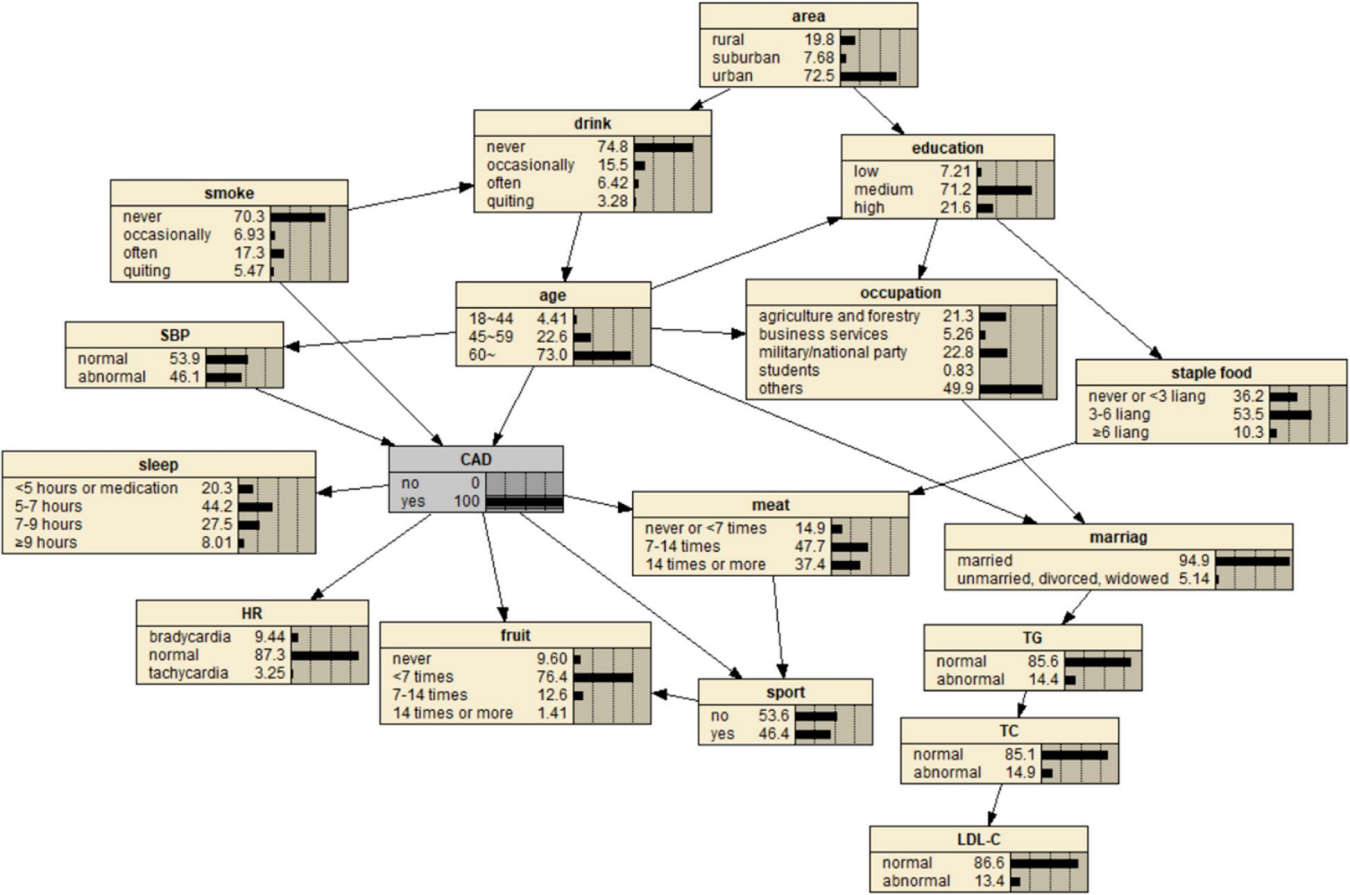
Diagnostic inference of BN model for CAD influencing factors

The diagnostic inference of the BN model for the influencing factors of CAD is shown in Fig. 12. The incidence probability of CAD is set to 100%, and it is found that in terms of age, the probability of people aged 18–44 years old decreases from 37.2% to 4.41%, and the probability of people aged 45–59 years old decreases from 23% to 22.6%. The probability of patients aged 60 and above increased from 39.8% to 73.0%, indicating that advanced age can significantly increase the risk of CAD. In terms of regions, the value of rural residents increased from 18.9% to 19.8%, that of suburban residents increased from 7.55% to 7.68%, and that of urban residents decreased, indicating that the probability of CAD in rural and suburban residents was higher than that in urban residents. The probability value of agriculture and forestry occupations increased to 21.3%, indicating that the occupational population has a higher risk of CAD. In terms of education level, the probability of college and above people decreased, and the probability of illiteracy and primary school, middle school, high school or secondary school increased, indicating that people with low education level were more likely to get sick. In terms of marital status, the value of married people increased from 83.1% to 94.9%, indicating that the CAD incidence probability of married people was higher than that of unmarried, divorced and widowed people. The probability of regular smokers increased from 13.8% to 17.3%, indicating that regular smokers had a higher risk of CAD. The probability of occasional drinking decreased, suggesting that moderate drinking may reduce the probability of disease. In terms of dietary habits, the probability value of people who do not eat or fruit intake is less than 7 times/week, meat intake is 7–14 times/week and less, and staple food intake is 3–6 two or more/day is increased, indicating that they are more likely to suffer from CAD. In terms of sleep time, the value of < 5 h/day or need drugs to help sleep increased from 11.1% to 20.3%, the value of 5–7 h/day decreased from 47.6% to 44.2%, the value of 7–9 h/day decreased from 36.7% to 27.5%, and the value of ≥ 9 h/day increased from 4.53% to 8.01%. The results showed that insufficient or too much sleep would increase the probability of CAD, and the effect of insufficient sleep might be greater than that of too long sleep. The value of abnormal systolic blood pressure increased from 27.2% to 46.1%, indicating that the increase of blood pressure will increase the incidence of CAD. At the same time, the probability values of no exercise, slow heart rate and abnormal TC, TG and LDL-C indexes also increased, indicating that CAD was more likely.

**Fig. 13:**
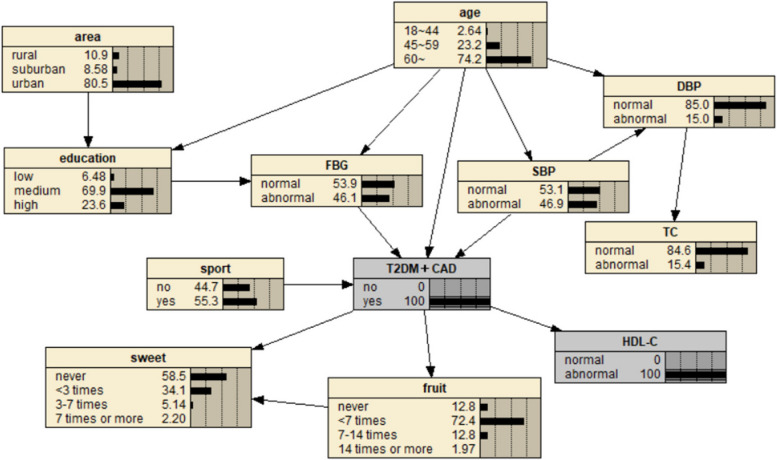
Diagnostic inference of BN model for comorbidity influencing factors

The diagnostic inference of the factors affecting comorbidity by BN model is shown in Fig. 13. The incidence probability value is set to 100%. It is found that in terms of age, the probability decreases from 46.4% to 2.64% for people aged 18 to 44, increases from 22.6% to 23.2% for people aged 45 to 59, and increases from 31% to 74.2% for people aged 60 and above. It showed that old age significantly increased the risk of comorbidities. The probability values in rural and suburban areas increased to 10.9% and 8.58% respectively, suggesting that the population living in rural and suburban areas had a higher probability of disease. Systolic blood pressure abnormalities increased from 23.5% to 46.9%, diastolic blood pressure abnormalities increased from 10.1% to 15%, indicating that hypertension can significantly increase the incidence of comorbidities. The value of abnormal fasting blood glucose increased from 17.2% to 46.1%, suggesting that DM may accelerate the course of coronary heart disease and increase the probability of comorbidity. In terms of exercise, the value of no exercise increased from 36.3% to 44.7%, indicating that lack of exercise can increase the risk of comorbidity. For total cholesterol and high-density lipoprotein cholesterol, the abnormal value increased by 0.5% and 13.4%, respectively, indicating that hyperlipidemia was one of the risk factors for comorbidities. In addition, the probability value of not eating or fruit intake less than 7 times/week, and not eating or sweet food intake more than 7 times/week also increased, indicating that the likelihood of comorbidities was also greater.

#### Sensitivity analysis

Sensitivity analysis is a method to quantify the degree of factor dependence in the BN model, which can reflect the quantization of target nodes caused by changes in local parameters of the network model, and then identify the sensitivity factors in the model. Through the forward reasoning of BN, sensitivity analysis of target variables "T2DM", "CAD" and "Comorbidity" can be conducted to obtain the influence of each factor on the outcome of the disease. The results of sensitivity analysis are expressed by the percentage of variance reduction, which can reflect the influence of specific variables on target variables. The larger the percentage of variance reduction value is, the greater the influence of input factors will be. The analysis results are shown in Table 12. As can be seen from the table, for T2DM, education level, age, region, and occupation ranking in the front in the influencing variables. For CAD, age, education level, occupation, fruit intake and SBP were more sensitive to CAD. For comorbidities, age, FBG, education level, fruit intake and SBP had significant effects on comorbidities. By focusing on the prevention and control of the above sensitive factors, the risk of disease can be effectively reduced.

**Table 12:** Sensitivity to target variable

T2DM		CAD		Comorbidity	
Variable	Variance reduction(%)	Variable	Variance reduction(%)	Variable	Variance reduction(%)
education	10.70	age	11.70	age	8.96
age	8.72	education	4.11	FBG	5.19
area	6.01	occupation	4.11	education	3.82
occupation	5.72	fruit	3.97	fruit	3.06
sweet	4.74	SBP	3.50	SBP	2.70
fruit	3.90	sleep	2.65	sweet	2.44
sport	1.58	Marriage	2.04	HDL-C	1.03
TG	0.39	sport	1.65	sport	0.27
drink	0.35	meat	1.58	DBP	0.23
HDL-C	0.25	HR	0.67	area	0.01
meat	0.22	drink	0.56	TC	0.00
LDL-C	0.15	staple food	0.27		
history	0.14	smoke	0.26		
staple food	0.11	TG	0.02		
HR	0.04	area	0.01		
smoke	0.02	TC	0.00		
		LDL-C	0.00		

## Discussion

In this study, we formulate BN models to investigate the relationships among influencing factors and the occurrence of T2DM, CHD and their comorbidities. Additionally, we employ these models to conduct predictive inference and etiological diagnosis of these diseases.

The results of multivariate Logistic regression analysis in this study showed that the probability of T2DM, CAD and their comorbiditis increased by 8.0%, 9.1% and 10.6% with each increase of one year of age, respectively. In BNs, age not only has a direct effect on disease, but also indirectly increases the risk of disease through variables such as education level, systolic blood pressure, occupation and FHx. From the results of parameter learning, it can be seen that for any disease, the elderly have the highest value of conditional probability, followed by middle-aged people and young people, which is consistent with the results of many studies [29–31]. There is no doubt about the harmful effect of old age on chronic diseases. On the one hand, it may be related to the aging of the body and the decline of physiological functions. On the other hand, people are exposed to more risk factors in the aging process, and chronic disease is the result of long-term accumulation of risk factors, when the accumulation of risk factors to a critical point, more likely to cause disease [32]. Therefore, we should focus on the health education of the elderly population, strengthen the comprehensive management and prevention of chronic diseases in the elderly, and promote healthy aging. In addition, there are gender differences in the prevalence of T2DM, and more men are diagnosed with T2DM globally, which is consistent with the results of this study. However, no significant relationship between gender and coronary heart disease and comorbidities was found in this study, suggesting that gender may not be a significant risk factor for the disease, and it needs to be combined with other risk factors to have an impact on the disease [33, 34].

The prevalence of DM among adults in rural China is rising faster than in urban areas, and the China Cardiovascular Disease Report also indicates that deaths from cardiovascular disease continue to be higher in rural China than in urban areas [35, 36]. The results of this study are consistent with the above studies, and the BN structure shows that in addition to the direct effect of region on T2DM and comorbidities, it can also be indirectly related to it through education level and occupation, and the indirect influence on CAD is mainly through alcohol consumption, age and systolic blood pressure. On the one hand, this may be related to the accelerated urbanization process, the improvement of rural living standards, the physical activity of workers is far less than before because of mechanized planting methods, and the unreasonable diet and poor lifestyle lead to the increase of obesity, dyslipidemia and other risk factors. On the other hand, the rural and suburban population has limited education level, low education level, long physical labor hours, poor medical technology and other factors, which are prone to chronic diseases. Therefore, we should strengthen the health education and publicity in rural and suburban areas, improve residents' awareness of chronic diseases, promote their healthy behavior, and continue to promote the basic medical and public health service capacity building of township health, so as to strengthen the prevention and control of chronic diseases in rural areas.

Moreover, we found that a high degree of education is associated with a low risk of T2DM, CAD and their comorbidities, and those with a low degree of education have a shallow understanding of the disease and a relatively weak awareness of self-prevention and health care, resulting in a higher prevalence of this population. At the same time, the higher the education level and the per capita income level of the family, the easier it is to get better medical conditions and social resources, and the stronger the sense of self-protection [37]. This further suggests that people with low education level are the key targets of health education on core knowledge of chronic diseases, which should be spread in multiple ways and channels based on the characteristics of the population, so as to improve the awareness rate of chronic disease prevention and control among the population.

Compared with unmarried, divorced and widowed people, married people have a higher probability of CAD. BN suggests that marital status is indirectly associated with coronary heart disease. Marital status affects people's quality of life, and its advantages and disadvantages are also related to human health. At the same time, diet, daily activities and other aspects will be affected, and the long-term past naturally has a negative impact on the body, while the positive marital status is the opposite [38].

We also founded that regular smokers have an increased risk of both T2DM and CAD, which is consistent with the results of other studies. The main mechanism of action is that smoking is associated with central obesity, oxidative stress and inflammation, and ultimately leads to insulin resistance and hyperglycemia, which then leads to coronary heart disease and T2DM [39–42]. Therefore, reducing tobacco use should be prioritized as an important public health strategy that will help prevent and control disease. Excessive alcohol consumption can lead to chronic disease, however, recent evidence suggests that light to moderate alcohol consumption is a protective factor against T2DM and cardiovascular disease, and the results of this study support this conclusion [43, 44]. The mechanism of how moderate alcohol consumption reduces the risk of disease remains to be elucidated, with research suggesting that moderate alcohol consumption may increase insulin sensitivity and reduce insulin resistance, can significantly reduce the risk of fatty liver disease, and is also significantly associated with better oral health [45].

Regarding dietary aspects, our study showed that people with meat intake less than 7–14 times/week had an increased probability of T2DM and CAD. This differs from previous research reports [46–49], and the reason for the divergent results may be that, following an early diagnosis, patients consciously decreased their meat intake by adjusting their diet. Consequently, the observed meat intake among patients was lower than that of the control group. In addition, the BN structure showed that meat can be indirectly associated with T2DM through fruit, further suggesting that the occurrence of disease is not entirely caused by meat itself, but may be related to dietary patterns. At the same time, people who do not eat or eat fruit less than 7 times/week have an increased probability of T2DM, CAD and their comorbiditis. Several studies have confirmed that fruits and vegetables can reduce the risk of T2DM and coronary heart disease CHD, which may be attributed to the substantial amount of dietary fiber, antioxidant vitamins, and folic acid present in vegetables, fruits, and other plant-based foods [50–53]. In addition, the study results found that intake of sweets was directly related to T2DM and comorbidities, and the probability of developing T2DM increased in people who did not eat sweets or those who consumed sweets for 7 or more times/week. Two kinds of extreme intake of sweets appeared. Analysis of the reasons for this result confirmed that high intake of sweets was associated with high risk of disease on the one hand; on the other hand, it may be due to the fact that the study subjects were all hospitalized patients. Most of them have been diagnosed with T2DM for a long time, so they pay attention to their own blood sugar control, thus reducing the intake of sweets. Moreover, patients in the comorbidity group pay more attention to their daily eating habits due to the combination of the two diseases, and rarely or almost do not eat sweets, thus failing to reflect the relationship between the real intake of sweets and the disease.

Exercise is considered a very important component of disease management, and some national and global guidelines recommend increased physical activity to prevent chronic disease. Our study also show that exercise can significantly reduce the probability of T2DM, CAD and their comorbidities, and the difference is more obvious with the older the age. Therefore, increasing physical activity is essential, and appropriate strategies should be developed to encourage patients to increase their exercise levels, especially those in middle age who are in the transition from adulthood to old age. At the same time, We have found that sleep time is a direct factor affecting coronary heart disease, and people who sleep less than 5 h per day or need medication to help sleep and people who sleep 9 h or more per day have a higher risk of CAD. A number of studies have also shown a U-shaped relationship between sleep time and major chronic diseases such as coronary heart disease, diabetes and stroke. Short and long sleepers were significantly associated with chronic disease [54, 55]. Both insufficient and excessive sleep can increase sympathetic nervous system activity and affect metabolic and inflammatory levels, and there is still a need for greater public awareness and sleep education, and for healthcare providers to strengthen discussions with patients about the importance of healthy sleep timing to help address the management and treatment of sleep timing disorders [56].

Hypertension can significantly increase the incidence of cardiovascular disease in diabetic patients, up to 75% [57]. At the same time, the risk of new T2DM in high-risk patients with cardiovascular disease and hypertension was 1.48 times higher than that in non-hypertensive patients, which was also confirmed by the results of this study [58]. Hypertension, DM and CAD are co-existing diseases that promote and influence each other. In the long-term blood pressure management of T2DM, CAD and their comorbid patients, various measures should be taken, including non-drug intervention prevention and control measures such as smoking cessation, weight reduction and diet adjustment, which are effective means to delay the development of the disease and prevent other adverse cardiovascular events. In addition, a number of clinical trials and epidemiological studies have confirmed that dyslipidemia is an important risk factor for T2DM and coronary heart disease [59–61]. The results of multi-factor logistic regression in this study showed that TC was a protective factor for CAD and comorbidities, which was obviously contrary to medical theory, and the reason may be related to inpatients taking lipid-lowering drugs on their own or receiving regular outpatient treatment, resulting in reverse regression coefficient symbols. In BN diagnostic reasoning, after CAD and comorbidities are set to "Yes", the probability values of abnormal TC, TG, HDL-C and LDL-C are all increased, precisely because BN can reflect the overall association between variables and is suitable for the study of non-independent complex network relationships between variables, and its reasoning is more in line with medical theory.

Logistic regression is a commonly used method to study the influencing factors of diseases. Although the OR value obtained can measure the degree of influence of various factors on diseases, it still has certain limitations, which cannot explore the specific relationship between influencing factors and diseases and the overall linkage effect of influencing factors. However, the advantages of BN can just make up for this limitation. It can intuitively display the direct or indirect relationship between diseases and risk factors through the topology structure, and can quantitatively describe the degree of correlation through the conditional probability value obtained by parameter learning. At the same time, from the perspective of medical biological effects, there is a complex network connection between diseases and factors, and between factors and factors, which can be manifested as a global linkage effect, in which the change of any controllable link will lead to the change of the overall effect. In addition, Bayesian network reasoning can handle incomplete data sets, predict the risk of disease occurrence when any node is known, conduct probabilistic reasoning based on the existing patient information, and constantly update the network probability with the increase of information. For the problems such as incomplete clinical information and deliberate concealment, which often occur in clinical diagnosis, it can still show strong empirical knowledge and effective reasoning function, which has unique advantages in medical application. From the validation effect of the three disease Bayes network models constructed in this study, the sensitivity and specificity are high, the AUC value is above 0.8, and the prediction performance is good, which indicates that the BN model has good application value in the field of chronic diseases such as T2DM and CAD, and also suggests the feasibility and applicability of BN in epidemiological research modeling.

The main shortcomings of this study are as follows: (1) The data came from the affiliated hospital and the second Affiliated Hospital of Guangdong Medical University, and the application and promotion of the research results of a single center has certain limitations;(2) The control group came from the physical examination center, and its age composition was quite different from that of the case group. The subsequent study will fully consider the influence of age on the occurrence of diseases, and include more comparable research objects to reduce bias.

## Conclusions

In this study, BN models of T2DM, CAD and their comorbidity influencing factors were constructed by tabu search algorithm. The prediction performance and reliability of BN models were good, and the prediction probability of disease occurrence was feasible and applicable, which could provide references for improving the prevention and control strategies of T2DM, CAD and their comorbidity and exploring the application of BN in the prediction and diagnosis of chronic diseases.

## Data Availability

The dataset utilized in the analysis of this study is available upon request from the corresponding author, Professor Ding Yuanlin, via email. As this study is a sub-project within the larger population disease research initiative of our research group, the comprehensive project is still ongoing. Consequently, the current research data is not ready for public release at this time. Readers interested in accessing the data may submit a formal data borrowing application to the corresponding author via email. Subject to reasonable requests, the author will facilitate the provision of the dataset associated with this study.
